# Robust pH Sensing Using a Graphene Oxide and Covalent Organic Frameworks Composite for Gastro‐esophageal Reflux Disease Diagnosis

**DOI:** 10.1002/adhm.202502106

**Published:** 2025-08-05

**Authors:** Ahmed H. M. Salem, Jianhui Zhang, Ashley Lam, Haowei Wang, Umber Cheema, Laurence B. Lovat, Manish K. Tiwari

**Affiliations:** ^1^ Nanoengineered Systems Laboratory UCL Mechanical Engineering University College London London WC1E 7JE UK; ^2^ UCL Hawkes Institute University College London London W1W 7TS UK; ^3^ UCL Medical Physics and Biomedical Engineering University College London London WC1E 7JE UK; ^4^ UCL Centre for 3D Models of Health and Disease UCL Division of Surgery and Interventional Science Faculty of Medical Sciences Charles Bell House University College London London W1W 7TY UK; ^5^ Centre for Precision Healthcare UCL Division of Medicine University College London London WC1E 6JF UK; ^6^ Manufacturing Futures Laboratory University College London London E20 2AE UK

**Keywords:** biocompatibility, covalent organic frameworks, gastro‐oesophageal reflux disease, graphene oxide, pH Sensing

## Abstract

Gastro‐esophageal reflux disease affects 15–20% of the global population and is commonly diagnosed via 24‐h ambulatory pH monitoring. However, current diagnostic tools, including catheter‐based systems and ingestible capsules, suffer from poor patient compliance and unreliable pH readings due to sensor degradation in acidic environments. To address this challenge, a robust, composite pH sensor is reported in this work by combining graphene oxide (GO) with covalent organic frameworks (COFs) coatings. Through interfacial polymerization and liquid‐phase exfoliation, the COF protective layer is covalently formed on GO sheets, preserving GO integrity in acidic conditions while enhancing electrochemical performance benefitting from the intrinsic porosity of COFs. The COFs nanochannels with sulfonic groups enable selective proton transport, resulting in high ion selectivity and sensitivity (≈43 mV pH^−1^, in pH 1–10). The sensor exhibits excellent linearity, stability, and high reproducibility in simulated gastric fluid. Furthermore, it resists biofouling from mucin‐rich environments and maintains performance under cyclic mechanical deformation. The GO/COFs composites also show biocompatibility with esophageal epithelial cells. These findings suggest that leveraging COFs as biocompatible protective coatings offers a promising strategy for enhancing the robustness and sensitivity of sensing materials in harsh environments and facilitates the future development of ingestible devices for continuous pH monitoring.

## Graphical abstract

A stable composite pH sensor is developed by coating graphene oxide (GO) nanosheets with covalent organic frameworks (COFs) to improve stability and sensitivity in acidic environments. The GO/COFs sensor demonstrates high selectivity, linearity, and biocompatibility, offering a promising strategy for continuous pH monitoring in gastro‐esophageal reflux disease diagnosis.

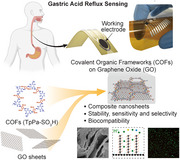

## 1 Introduction

Gastro‐esophageal reflux disease (GERD) is a common digestive condition where gastric acid rises into the esophagus, causing symptoms such as heartburn, the most sensitive and specific symptom. Patients often report a burning sensation in the chest and an unpleasant taste in the mouth, with an estimated prevalence of 15–20% in the general population.^[^ ^1^ ^]^ Diagnosis involves ≈24 h ambulatory pH and impedance monitoring to identify reflux triggers, enabling lifestyle changes to reduce reflux events. Reflux composition, including ingested food and drink, acid, stomach enzymes like pepsin, and bile, primarily causes laryngeal damage.^[^ ^2^ ^]^ This also affects sensors used for GERD diagnosis, such as the Bravo Capsule or catheter‐based sensors, leading to discrepancies in reflux event counts and recorded pH values.^[^ ^3^ ^]^ Additionally, current diagnostic methods are poorly tolerated due to their size and affixation method, highlighting the need for miniaturized, biocompatible, flexible, and cost‐effective sensors.^[^ ^4^, ^5^, ^6^, ^7^, ^8^, ^9^ ^]^

Modern electrochemical sensing and biosensing technologies often use printed electrodes arranged in 2‐ or 3‐electrode systems, valued for their simplicity, miniaturization potential, low cost, and suitability for mass production.^[^ ^10^ ^]^ Electrode performance depends on material modifications, which influence sensitivity, repeatability, stability, and selectivity. Metal oxides such as iridium oxide and zinc oxide are commonly used due to their high sensitivity and biocompatibility.^[^ ^11^, ^12^ ^]^ However, iridium oxide's high cost limits large‐scale applications^[^ ^13^ ^]^ while zinc oxide's water solubility in acidic conditions affects stability outside neutral pH.^[^ ^14^ ^]^

Polymer‐based sensors, such as polyaniline and its composites, achieve near‐Nernstian responses (−58 mV pH^−1^) but have biocompatibility concerns due to cytotoxic precursor materials such as aniline, aniline hydrochloride, and ammonium persulfate.^[^ ^11^, ^15^ ^]^ Their sensing range is typically limited to above pH 2, leading to inaccuracies in gastric acid detection, and they often require a proton‐exchange membrane like Nafion 115, which raises cytotoxicity concerns due to its fluoride content.^[^ ^15^ ^]^ Composite sensors using polypyrrole offer better conductivity and pH responsiveness, but also introduce cytotoxicity concerns depending on synthesis method and residual monomer content.

Graphene oxide (GO) is a widely used, biocompatible, pH‐responsive material, known for its high concentration of oxygen‐containing functional groups, tunable functionalization, and excellent biocompatibility. However, under harsh acidic conditions, GO can lose these functional groups from partial reduction, leading to reduced pH sensitivity and inconsistent performance.^[^ ^16^, ^17^ ^]^ Further, at low pH, GO aggregates into a sandwich‐like structure with water molecules, while at high pH, it dissolves like a regular salt, leading to instability and unreliable sensing.^[^ ^17^ ^]^ These limitations make GO unsuitable for continuous GERD monitoring, highlighting the need for more stable alternatives.

Several additives have been explored to address the limitations of GO, including tannic acid, Nafion, ruthenium dioxide, and various conducting polymers.^[^ ^18^, ^19^, ^20^, ^21^, ^22^ ^]^ However, many of these materials suffer from drawbacks such as poor stability in highly acidic environments, high cost, or potential cytotoxicity, which limit their applicability in biomedical contexts.^[^ ^18^, ^19^, ^21^ ^]^ Recent advances have introduced covalent organic frameworks (COFs), a class of porous nanomaterials with high surface area, excellent biocompatibility, and numerous electroactive sites, enhancing sensitivity.^[^ ^10^, ^23^ ^]^ Some imine COFs can function as 2D graphene‐like nanosheets and offer intrinsic acid/base stability due to irreversible tautomerization.^[^ ^24^ ^]^ It has been shown that GO's pH sensitivity for example arises from protonation and deprotonation of functional groups—amine, carboxyl, and hydroxyl.^[^ ^25^, ^26^ ^]^ Triformylphloroglucinol, used by Kandambeth *et* al. for acid‐stable COFs, contains hydroxyl and formyl groups.^[^ ^24^ ^]^ When combined with p‐phenylenediamine (PDA), which has highly reactive amino groups, COFs form with inherent protonation and deprotonation sites. Variants of PDA, such as 2,5‐diaminobenzoic acid (with a carboxyl group) or 2,5‐diaminobenzene‐1,4‐disulfonic acid (with sulfonic groups), can serve as specialized linkers in COF synthesis. Electrochemical COF‐based sensors have been reported in the literature for the detection of cardiac troponin,^[^ ^27^ ^]^ Pb^2+^,^[^ ^28^ ^]^ and even pH.^[^ ^29^ ^]^ However, the strategies employed to address their intrinsically low conductivity often involve the incorporation of nanoparticles or cytotoxic materials, limiting their suitability for in vivo applications.

Here, we report GO combined with directionally‐stacked COFs formed via interfacial synthesis to enable π–π stacking, for in vivo pH sensing applications.^[^ ^30^ ^]^ The COF layer preserves the structural integrity of GO while introducing accessible active sites for protonation and deprotonation, crucial for pH sensitivity. COFs offer three major advantages over alternative sensing materials such as pristine GO or metal oxides. First, high chemical tunability, enabling the incorporation of pH‐sensitive or proton‐selective groups. Second, intrinsic ordered porosity and high surface area, promoting efficient ion transport and dense functionalization. Third, excellent stability in aqueous and acidic environments. The low conductivity of COFs was mitigated by GO, which became conductive through high‐temperature synthesis, enhancing electrochemical response.^[^ ^31^ ^]^ The 2D stacked nanoporous structure act as a network of nanochannels, increasing the capacitive response and contributing to a well‐defined electrical double layer.^[^ ^32^ ^]^ Furthermore, sulfonic acid groups (─SO_3_H) from COFs, akin to those used in Nafion, introduce selective proton conductivity, enabling a selective double‐layer response by blocking interfering ions.^[^ ^32^ ^]^ The hydrophilic and porous nature of the COF coating also offers inherent anti‐biofouling properties that limit mucin and protein adsorption.^[^ ^33^ ^]^ We demonstrated that the GO/COF sensor remained stable in mucin‐rich simulated gastric fluid, exhibited excellent electrochemical stability after enzymatic exposure, and maintained structural and electrochemical integrity under repeated mechanical bending and UV sterilization. Finally, biocompatibility of the composite was confirmed through in vitro cytotoxicity assays using esophageal epithelial cells, supporting its suitability for gastric and esophageal sensing applications.

## 2 Results and Discussion

### 2.1 Design of Robust pH Sensor

**Figure 1:**
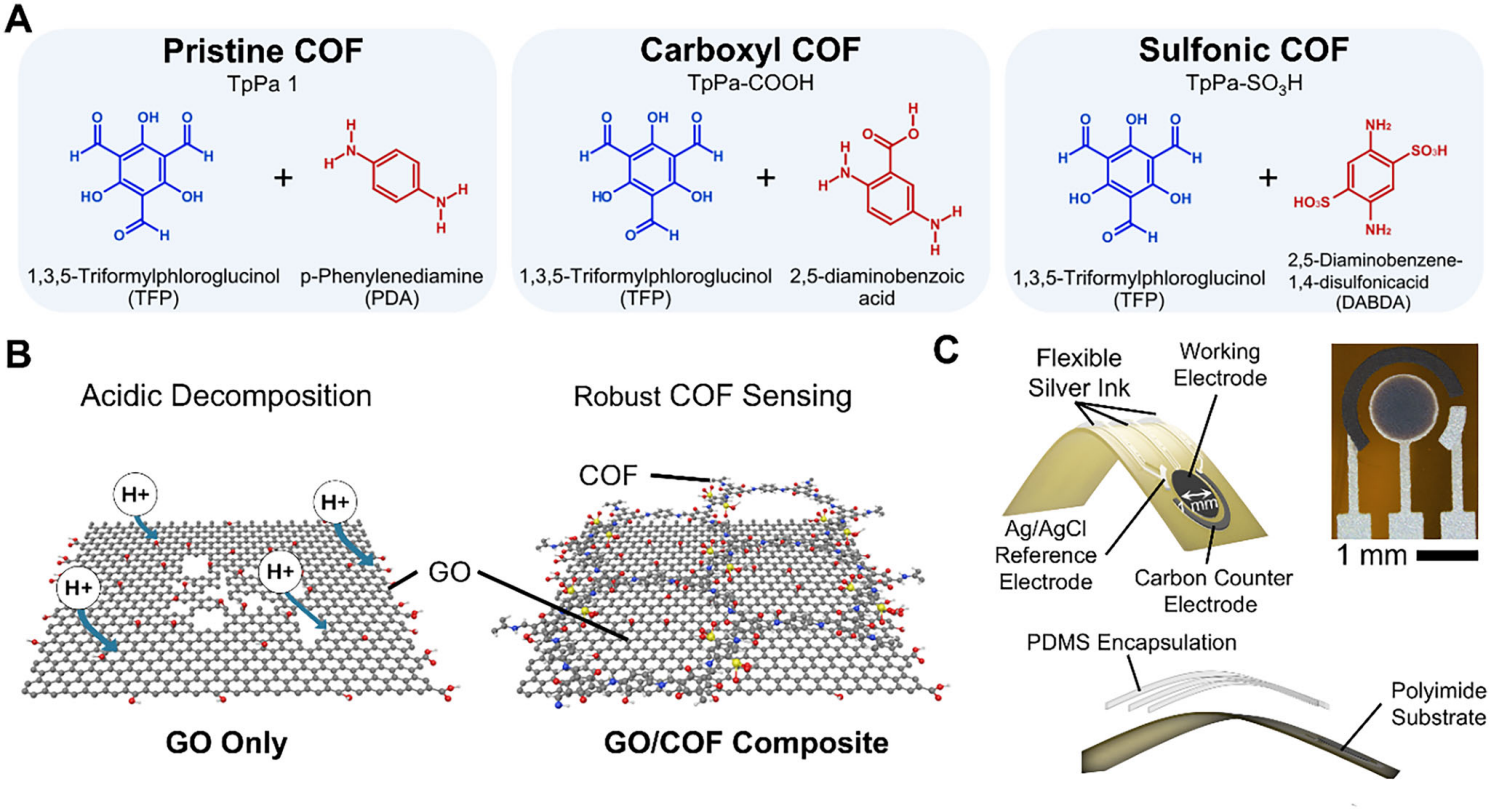
Design of robust pH sensor using a GO/COF composite. A) Chemical structures of pristine COF (TpPa‐1), carboxyl COF (TpPa‐COOH) and sulfonic COF (TpPa‐SO_3_H) showing the difference in functional groups present. B) Schematic (left) of protonation and acidic instability of pure GO leading to poor stability. (right) Schematic showing the formation of COF nanopores on the GO, which increases surface area, functional sites, and stability. C) Schematics and image of the printed 3‐electrode configuration used for electrochemical characterization.

Three acid‐stable COFs were combined with GO to form composites and evaluated for their pH response in a gastric acid simulant. These COFs—pristine COF, carboxyl COF, and sulfonic COF—were named according to their functional groups (**Figure** **1**A). The inherently low conductivity of COFs was addressed by incorporating GO, which, upon reduction via high‐temperature synthesis and chemical treatment, became conductive, enhancing the electrochemical response. The formation of 2‐D stacked nanopores, which duly serve as a protective layer and as nano‐channels, would prevent acidic decomposition and increase the surface area, eliciting a significantly larger electrical double layer (Figure 1B). The composites were deposited onto the working electrode of a flexible three‐electrode printed sensor, consisting of an Ag/AgCl reference electrode, a carbon counter electrode, and an Ag‐based flexible ink working electrode (Figure 1C). Exposed silver area was encapsulated with PDMS along the tracks and around the working electrode using direct‐write printing with in situ heat curing (Figure S1, Supporting Information).

### 2.2 Characterization of GO/COF Composite

**Figure 2:**
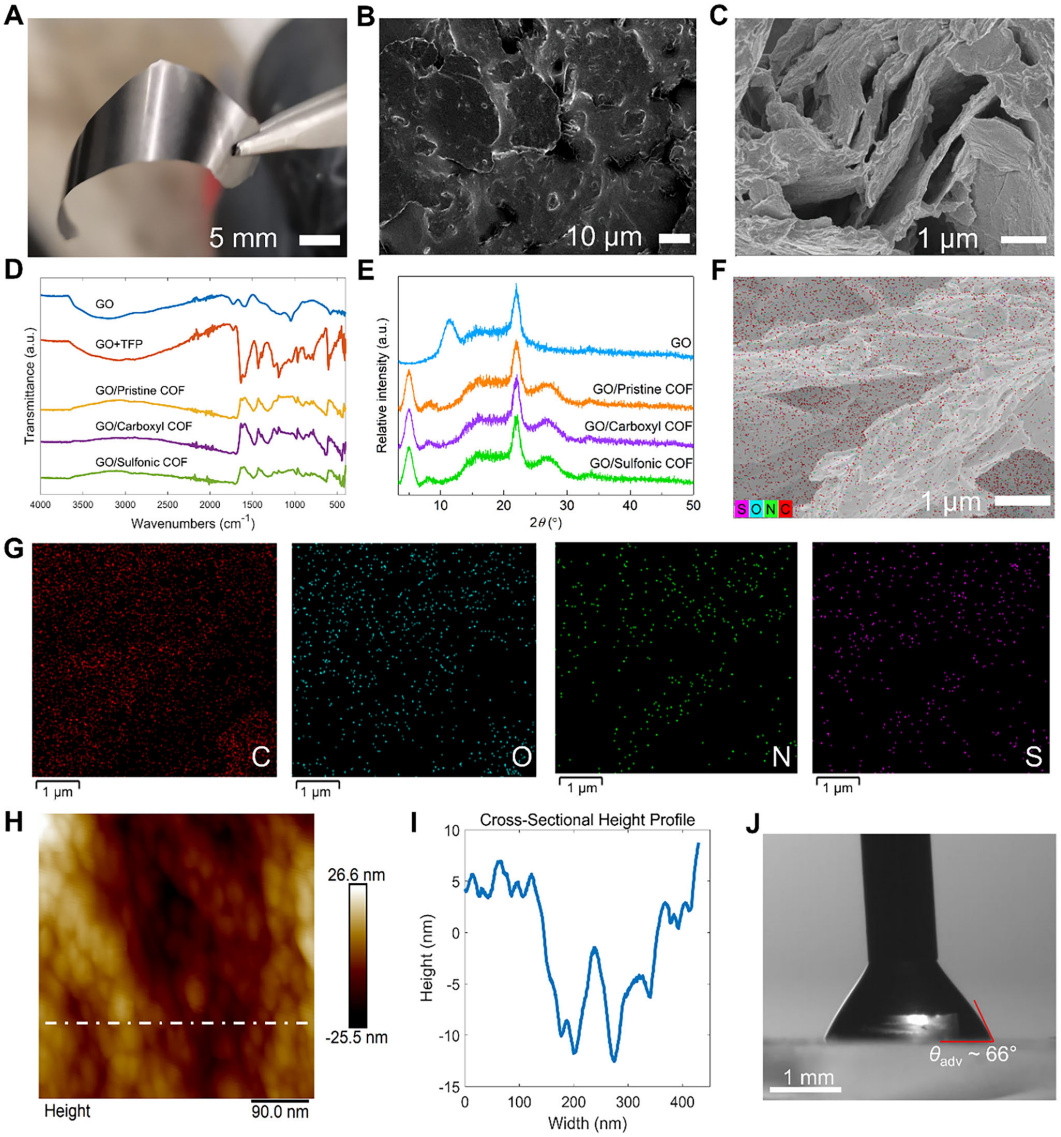
Characterization of GO/COF composite. A) Photograph of peeled GO/Sulfonic COF composite film showing a metallic‐like luster and mechanical integrity. B) SEM image of the stacked structure of the GO/Sulfonic COF film prior to exfoliation. C) Cross‐sectional SEM image following liquid exfoliation showing the layers of the flakes. D) FTIR spectra of materials throughout GO/COF synthesis process E) XRD patterns of GO and GO/COF composites, confirming successful synthesis and crystallinity of the materials. F) EDS mapping of elemental composition on sulfonic COF SEM image. G) EDS major elements detected and their locations: carbon, oxygen, nitrogen, and sulfur. H) AFM image of the sample following liquid exfoliation. I) Height profile corresponding to the white dashed line in (H). J) Advancing contact angle (θ_adv_) of GO/COF sprayed on glass showing hydrophilic properties.

**Figure** **2**A presents a free‐standing film of GO/COF with a metal‐like luster after being peeled from a glass substrate. All three synthesized GO/COF materials exhibited similar optical properties when prepared using the same interfacial synthesis method detailed in Figure S2 (Supporting Information). The formation of a free‐standing film with black gloss indicates that the formation of COF between GO layers bonded GO flakes together. This was further confirmed by scanning electron microscopy (SEM) images before and after liquid exfoliation (Figure 2B,C). Figure 2B shows the structural integrity of the GO/COF coatings prior to liquid exfoliation. After exfoliation (Figure 2C), the GO/COF composites formed micro‐sized flakes with a multi‐layer stacking structure, highlighting the structural integrity and layer separation achieved through liquid exfoliation, which likely enhances active sites for pH detection. Fourier‐transform infrared spectroscopy (FTIR), as shown in Figure 2D, revealed COF formation throughout the synthesis process showing characteristic peaks at 1306 cm^−1^ indicating (C―N) stretching and (C═C) stretching ≈1617 cm^−1^, (details in Figure S3, Supporting Information) aligning with literature.^[^ ^24^, ^32^, ^34^ ^]^ The crystalline structure and the composition of the composites were confirmed by X‐ray diffraction (XRD), showing patterns consistent with previously reported results,^[^ ^34^ ^]^ as illustrated in Figure 2E. The energy dispersive X‐ray spectroscopy (EDS) mapping, as shown in Figure 2F, was also used to confirm the formation of the sulfonic COF on GO nanosheets indirectly. The EDS shows that the major elements detected are carbon, oxygen, nitrogen, and sulfur following intense washing of unreacted linkers with chloroform, indicating the successful bonding of the sulfonic linkers (Figure 2G). On the surface of the flakes, a typical nanohierarchical morphology of COFs with small bumps (≈45 nm) was found through atomic force microscopy (AFM) (Figure 2H,I), which showed similar patterns as pure smooth COF coatings on glass in literature,^[^ ^35^ ^]^ suggesting successful coating of COFs on GO sheets without defects. Both the inherent porosity of the COF and the enhanced surface roughness of the COF layer with nanohierarchical texture, as confirmed by XRD (Figure 2E) and AFM (Figure S4, Supporting Information), respectively, led to the increase in surface area of GO/COF. Figure 2J shows the advancing contact angle (*θ_adv_*), and the wetting properties of the exfoliated material indicate a predominantly hydrophilic nature (≈66°), which may contribute to biofouling resistance.^[^ ^33^ ^]^

### 2.3 Electrochemical Performance

**Figure 3:**
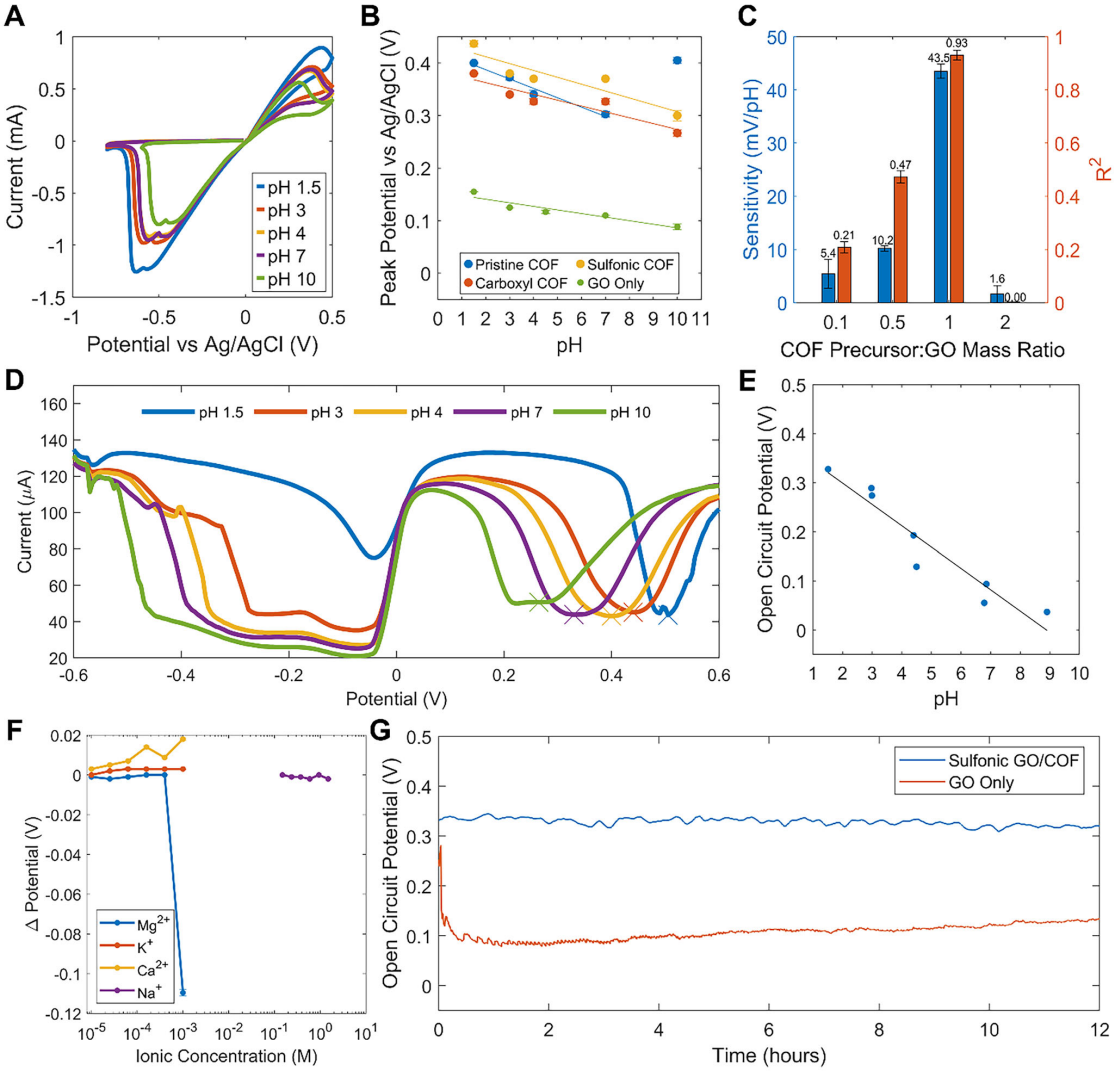
Electrochemical performance. A) Cyclic voltammetry (CV) response of Sulfonic COF composite in different pHs. B) Calibration curve oxidative peak against pH derived from CV scans of the different COFs and GO. C) Open circuit potential (OCP) sensitivity and linearity of Sulfonic COF composite in different mass ratios between TFP (COF precursor) and GO (*n* = 3). D) Differential Pulse Voltammetry (DPV) response of Sulfonic COF. E) OCP pH calibration curve. F) Fixed‐interference method selectivity against Mg^2^ *^+^*, K^+^, Ca^2+^, Na^+^ ions using OCP. G) Time‐series measurements of GO/Sulfonic COF compared to pure GO in pH 1.5 gastric acid simulant.

Cyclic voltammetry (CV) scans (**Figure** **3**A) revealed a pH‐sensitive response, particularly in the oxidative peak. The responses of the three GO/COF composites across different pH levels were plotted (Figure 3B), demonstrating that the sulfonic GO/COF composite exhibited the highest sensitivity across pH 1–10. The sulfonic COF possesses a high density of ordered 1D nanochannels, with sulfonic acid groups (─SO_3_H) on the pore walls. These groups function as proton sources, facilitating proton hopping via the deprotonated sulfonate groups (─SO_3_−) and their subsequent re‐protonation. This mechanism enables a selective buildup of H+ ions, contributing to increased capacitance due to the accumulation of protons in the electrical double layer. The COF precursor:GO mass ratio was varied to optimize the composite formulation, with open circuit potential (OCP) measurements across pH 1–10 confirming that a 1:1 ratio yielded the best combination of high sensitivity and strong linearity (Figure 3C). Linearity and reproducibility were quantitatively assessed using the coefficient of determination (*R* ^2^), calculated from linear regression fits during both increasing and decreasing pH sweeps on the sensors (*n* = 3). A high *R* ^2^ value indicated strong linear correlation and good repeatability of the sensor's behavior. The results indicate that, following deoxygenation, GO alone does not exhibit a pH response as predicted.^[^ ^26^ ^]^ Instead, increasing COF content enhances pH sensitivity up to a critical point, beyond which excessive COF reduces conductivity and diminishes the response.

Differential pulse voltammetry (DPV) measurements, performed using a three‐electrode configuration with an amplitude of 50 mV and a pulse width of 0.2 s, exhibited enhanced linearity across the pH range of 1–10. This improvement is attributed to a higher signal‐to‐noise ratio, achieved by minimizing background charging and suppressing non‐faradaic capacitive currents. Clear and progressive shifts in peak current responses were observed with varying pH, further confirming the sensor's sensitivity and electrochemical stability (Figure 3D). A corresponding calibration curve is provided in Figure S5 (Supporting Information). OCP calibration curves further demonstrated higher pH sensitivity than those obtained through voltametric techniques (Figure 3E). pH selectivity measurements, conducted using the Fixed‐interference Method (FIM) in a simulated gastric acid environment, demonstrated high selectivity toward commonly ingested interfering cations, including magnesium (Mg^2+^), potassium (K^+^), calcium (Ca^2+^) and sodium (Na^+^) ions (tested as their chloride salts e.g MgCl_2_, KCl) (Figure 3F). The FIM results confirmed the sensor's strong preference for the primary H+ ion over these potential interferents, while maintaining relevance to real‐world gastric conditions, where multiple ionic species coexist.^[^ ^36^ ^]^ Among these, magnesium ions exhibited the most significant potential shift, particularly at concentrations exceeding 10^−3^ m. However, this effect is unlikely to compromise sensor performance in gastric acid sensing, as the typical dietary intake of magnesium is relatively low, resulting in only trace amounts being present in the stomach.^[^ ^37^ ^]^

Compared to pure GO, the sulfonic GO/COF composite exhibited enhanced electrode stability under acidic conditions in simulant gastric acid (Figure 3G). It also maintained high stability across a broad pH range (1.5–10) over a 12‐h testing period (Figure S6, Supporting Information). This can be described by Equation (1), which details that as a polarizable electrode reaches steady state equilibrium, it exhibits the same potential as a capacitor that is charged galvanostatically.^[^ ^38^ ^]^

(1) $$E=E_{0}+i\left(R+t/C\right)$$

here, *E* is the electrode potential, *i* is the current, *R* is the bulk resistance of the electrode, *t* is time, and *C* is the electrode capacitance at low frequencies. The time dependence of the electrode potential (potential temporal drift) is then given by Equation (2).^[^ ^38^ ^]^

(2) Potentialdrift=ΔE/Δt=i/C

Equation (2) suggests that a sufficiently large electrode capacitance is necessary for potential stability. The increased capacitance can come from the increased surface area.

Capacitive measurements were performed using an impedance analyzer on interdigitated electrodes coated with GO/Sulfonic COF, across a frequency range of 10^2^ – 3 × 10^5^ Hz in different pH solutions, revealing a highly linear pH response (Figure S7, Supporting Information). This suggests that H+ ions are the primary contributors to the observed capacitance behavior, likely due to the presence of ─SO_3_− groups, which facilitate selective proton hopping. This also revealed that the material can be used to sense pH without the need for a reference Ag/AgCl reference electrode and be used as a purely capacitive sensor.

### 2.4 Sterilization Treatment and Biofouling Resistance

To assess the compatibility of the sensor with standard clinical sterilization procedures, we evaluated its electrochemical performance before and after two commonly used methods: ultraviolet (UV) irradiation and autoclaving. As shown in Figure S8 (Supporting Information), CV measurements revealed only minor changes in both anodic and cathodic peak currents and negligible shifts in peak potentials following UV exposure. This indicates that the sensor retained stable electrochemical behavior under this mild sterilization protocol. In contrast, autoclaving caused a substantial increase in current response, likely due to partial delamination of the PDMS encapsulation layer, which exposed the silver interconnects to the electrolyte (see Figure S8D, Supporting Information). While the sensor shows good compatibility with UV sterilization, future designs may require improved encapsulation strategies to ensure stability under high‐temperature, steam‐based sterilization.

**Figure 4:**
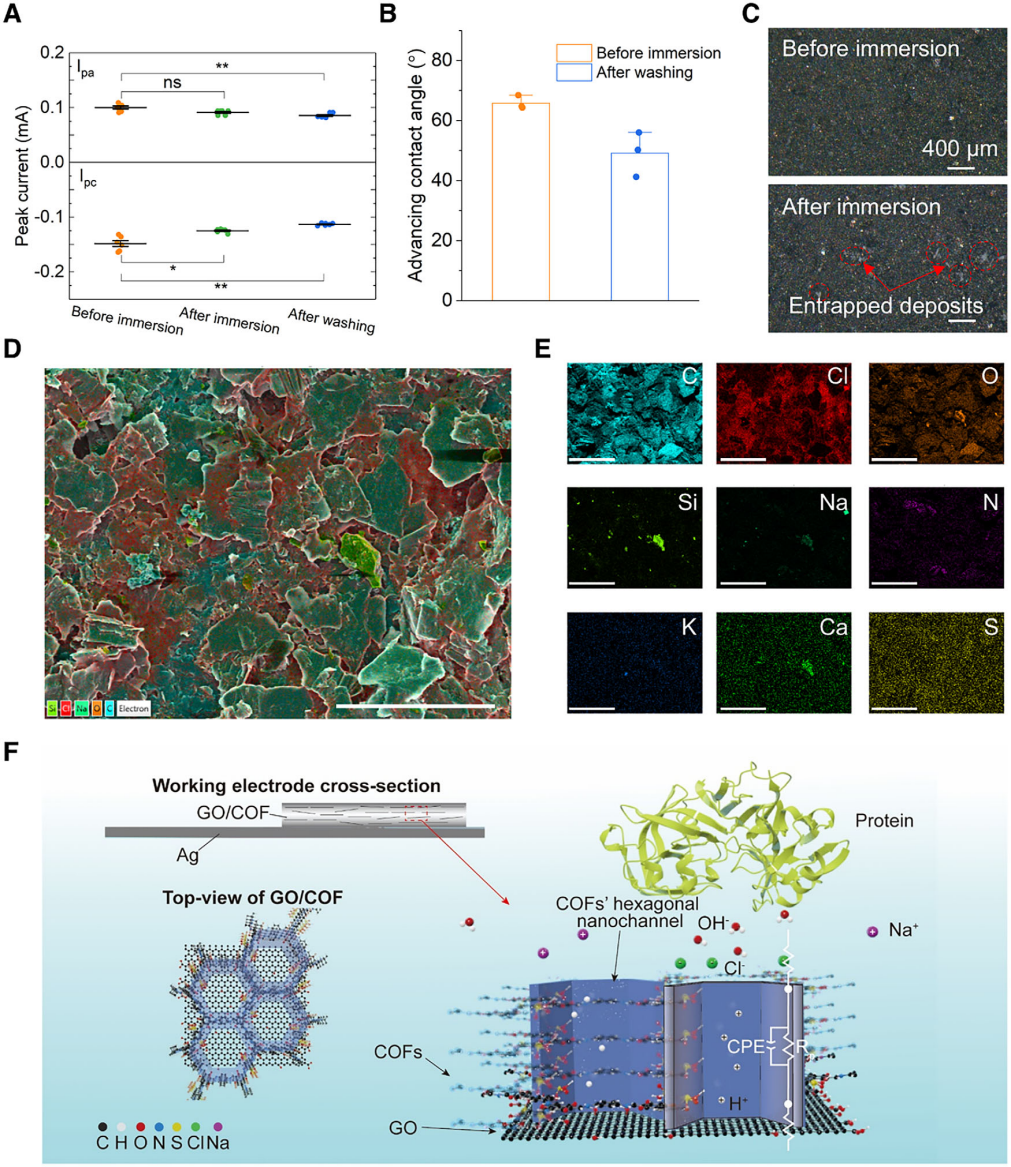
Bio‐fouling test in mucin‐ and enzyme‐rich simulated gastric fluid (SGF) and sensing mechanism. A) Anodic (I_pa_) and cathodic (I_pc_) peak currents extracted from CV measurements before immersion, after 24 h exposure in SGF, and following PBS rinsing. Statistical significance is indicated as follows: ns (not significant), *p* > 0.05; ^*^ *p* ≤ 0.05; ^*^ *p* ≤ 0.01. B) Advancing contact angle measurements of water droplets on the sensor surface before and after SGF immersion. C) Optical microscopy images of the GO/COF electrode surface before and after 24 h SGF exposure, showing no major structural degradation. Residual entrapped deposits from dried SGF are visible within microscale gaps between particles. D) SEM–EDS elemental mapping of the COF/GO sensor surface after 24‐h immersion in simulated gastric fluid (SGF). Scale bar: 100 µm. E) Individual EDS maps of detected elements, including C, Cl, O, Si, Ca, N, S, Na, and K, showing that SGF‐derived residues were detected only in spatially limited regions between particles, indicating localized rather than widespread surface fouling. Scale bar: 100 µm. F) Schematic illustration of the interfacial structure, pH‐selective sensing mechanism, and equivalent circuit model of the GO/COF composite. The lamellar structure of the GO/COF composite facilitates directional proton (while balls, H^+^) diffusion through the interconnected 2D nanochannels formed by the COF layer, while the GO sheet provides a conductive backbone for efficient charge transport. The shaded purple walls represent the aligned nanochannels formed by the packed COF in cross‐section. The intrinsic porosity of the COF not only increases the density of ion‐accessible sites but also supports the formation of a well‐defined electrical double layer, thereby enhancing the capacitive response. Sulfonic acid groups integrated into the COF selectively attract protons while repelling interfering ions such as Na^+^, Cl^‐^ and biomolecular contaminants like pepsin protein.

To evaluate the sensor's stability and resistance to biofouling under physiologically relevant conditions, we immersed the GO/COF electrode in a simulated gastric fluid (SGF) formulated with 0.32% (w/v) pepsin, 0.5 mg mL^−1^ mucin, 0.1 mg mL^−1^ bile salts, and 15 µg mL^−1^ lipase for 24 h at 37 °C.^[^ ^39^, ^40^, ^41^, ^42^ ^]^ As shown in **Figure** **4**A, the anodic peak current (I_pa_) remained statistically unchanged after 24‐h exposure, while the cathodic peak current (I_pc_) showed a slight decrease, indicating minor surface interaction without significant deterioration of electrochemical activity. Both peak currents decreased more noticeably after PBS rinsing. This may be attributed to the partial detachment of GO/COF particles during the cleaning process, which slightly reduced the active surface area. Contact angle measurements (Figure 4B) revealed a modest increase in surface hydrophilicity following SGF exposure, with the advancing contact angle decreasing to ≈50°. This change likely resulted from hydrophilic biofouling components—such as proteins and bile salts—becoming entrapped in the microscale gaps between particles (see Figure 4C). Further support for this interpretation was provided by SEM–EDS mapping (Figure 4D,E), which revealed localized detection of elements such as N, Ca, K, and Na. These elements were characteristic of SGF‐derived residues. These findings indicated that although biofouling components could adsorb between surface particles, their distribution was limited and did not extensively alter the sensing performance. The overall electrode structure remained intact. However, the size of these residual molecules is orders of magnitude larger than the COF nanochannels and does not obstruct proton‐selective pathways. Consequently, no significant degradation in pH sensing performance was observed. To minimize particle detachment and inter‐particle voids, future designs may adopt a layered nacre‐inspired architecture.^[^ ^43^ ^]^ This could be achieved by assembling GO/COF nanosheets into a lamellar configuration and binding them via bioadhesive agents such as dopamine or tannic acid, enhancing both mechanical cohesion and substrate adhesion.

### 2.5 Sensing Mechanism

To gain further insight into the underlying sensing mechanism, a schematic representation of the GO/COF interface structure was developed (Figure 4F). The diagram illustrates how the lamellar assembly of GO and sulfonic acid‐functionalized COF enables directional proton transport through confined hexagonal nanochannels, while simultaneously repelling interfering ions and biomolecular contaminants. In addition to ion selectivity, the COF layer also acts as a protective barrier that stabilizes the sensing interface under strongly acidic conditions, ensuring reliable performance even in harsh environments. This structural arrangement, combined with the electronic conductivity of GO and the selective ion affinity imparted by ─SO_3_^−^ groups, explains the strong pH‐dependent capacitive behavior observed in the experiments.

This trend was further supported by electrochemical impedance spectroscopy (EIS) measurements in a two‐electrode setup (Figure S9, Supporting Information), where the fitted equivalent circuit consists of a solution resistance (R_s_), a polarization resistance (R_p_), and a constant phase element (CPE). Although the capacitive behavior in this configuration was less ideal—due to interfacial polarization and asymmetry between electrodes—the R_p_ parameter showed a consistent increase with pH. This was attributed to reduced proton mobility and weakened ionic coupling within the COF layer at higher pH, confirming that the sensing mechanism was primarily governed by selective proton transport across the GO/COF composite interface.

### 2.6 Mechanical Durability

**Figure 5:**
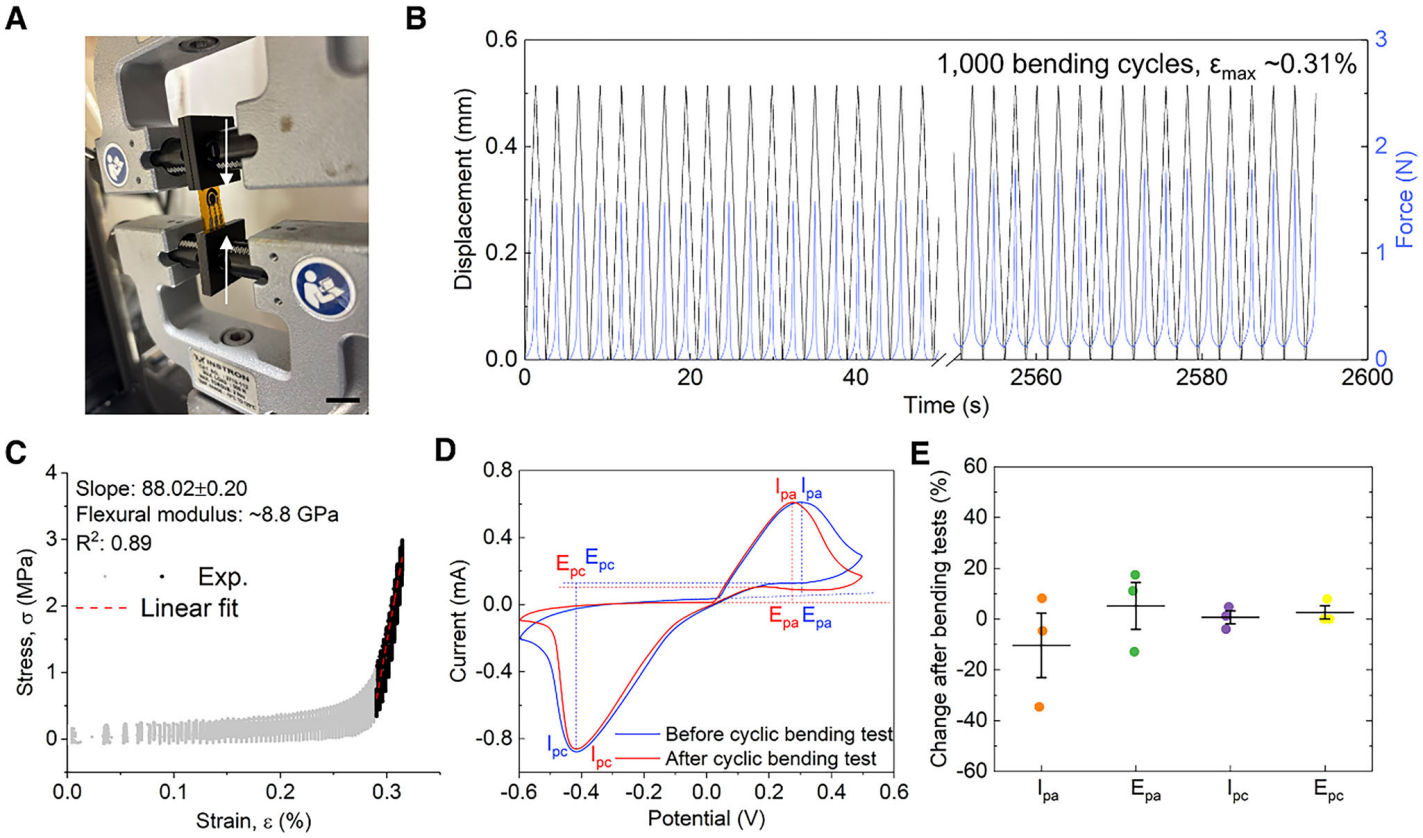
Mechanical stability and electromechanical performance of the flexible sensor under cyclic deformation. A) Photograph of the sensor under end‐to‐end compression on an Instron machine, following a bending setup with both ends clamped. Scale bar: 2 cm. B) Force–displacement curve over 1000 compression–bending cycles demonstrating mechanical repeatability. C) Calculated stress and strain rate based on the geometric parameters of the sensor, with linear elastic region highlighted (black) and fitted (red dashed line). D) Cyclic voltammetry curves acquired before and after cyclic mechanical testing, indicating electrochemical stability. I_pc_ and I_pa_ show the peak cathodic and anodic current, respectively. E_pc_ and E_pa_ denote the peak cathodic and anodic potential, respectively. E) Changes of anodic and cathodic peak potentials and currents after cyclic bending tests.

To evaluate the mechanical robustness and electrochemical stability of the sensor under physiologically relevant cyclic deformation, we performed compression–bending tests using an Instron machine configured in a double‐clamped axial compression setup, which mimics the repeated peristaltic motions encountered in esophageal environments (**Figure** **5**A). This durability is aided by the PDMS encapsulation, which forms a seal that ensures conformal encapsulation of the electrodes and a reservoir which promotes uniform coverage of the working electrode by the GO/COF composite (Figure S10A,B, Supporting Information). PDMS was selected for its flexibility, biocompatibility, and established use in ingestible electronics. Although not a perfect moisture barrier, it offers excellent electrical insulation and is well‐suited for soft bioelectronic applications requiring conformal encapsulation.^[^ ^12^ ^]^ In our tests, no visible degradation or delamination was observed during in vitro immersion and handling, supporting its suitability over the intended diagnostic timescale.

Over 1000 deformation cycles, the sensor exhibited a small change in the force–displacement profile (Figure 5B), indicating excellent mechanical resilience. Based on the geometry of the sensor, we calculated the surface strain and stress during bending (Figure 5C). The linear elastic region was identified via regression fitting, yielding an effective flexural modulus of ≈8.8 GPa, which falls within the expected range for PI‐based multilayered systems.^[^ ^44^ ^]^ The maximum strain applied (≈0.3%) remains well within the elastic limit of the device structure, ensuring durability under repeated bending. Electrochemical performance was further evaluated before and after cyclic deformation by CV, which revealed negligible changes in redox peak positions (Figure 5D), confirming that the electrode interface remained intact. Microscopic inspection of the working electrode after 1000 bending cycles (Figure S10C–F, Supporting Information) revealed slight changes in surface morphology, including partial detachment of surface‐deposited nanoparticles. These microstructural disruptions can affect the local electron transfer kinetics and interfacial contact area, potentially affecting redox reactions. Both the anodic peak current (I_pc_) and potential (E_pc_) exhibited moderate variations (20–30%) after cyclic deformation (Figure 5E). In contrast, the cathodic response remained relatively stable. This suggests that, the oxidation process is more surface‐sensitive, likely due to its greater dependence on the exposed nanoparticle surface, whereas the reduction pathway remains intact, possibly involving more stable internal conduction pathways. These results validate the sensor's capacity to operate reliably in dynamic gastrointestinal conditions without mechanical or functional degradation.

### 2.7 Biocompatibility

**Figure 6:**
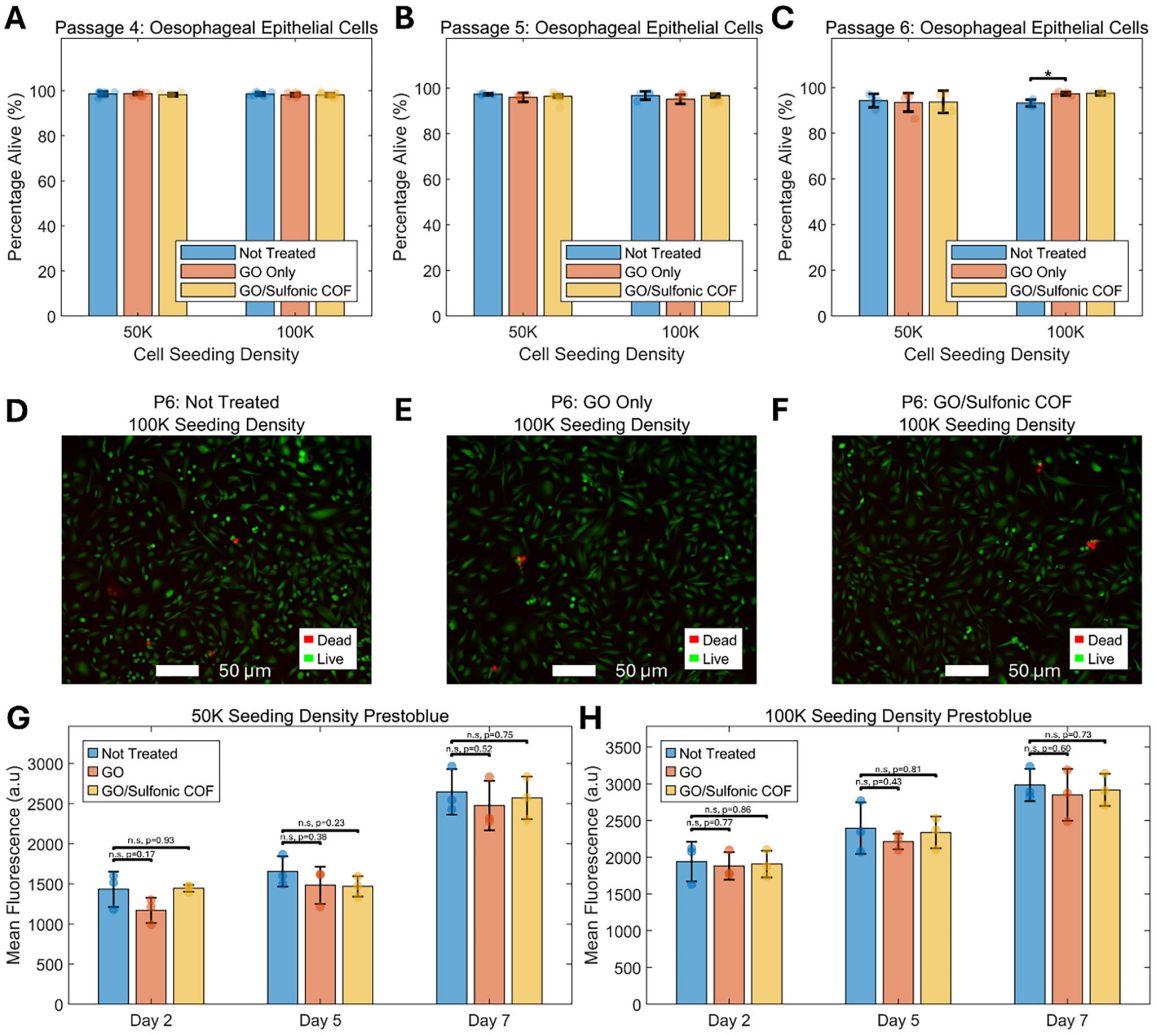
Biocompatibility of the sensing materials. Live‐dead cytotoxicity results for HEsEpiC cells in untreated samples, graphene oxide (GO), and GO/COF composite at A) passage 4, B) passage 5, and C) passage 6, expressed as the percentage of live cells (calcium green‐stained) relative to total cells, including ethidium homodimer‐stained dead cells. Representative fluorescent images of HEsEpiC cells at D) passage 6 with a seeding density of 100K are shown for untreated samples, E) GO, and F) GO/Sulfonic COF composite. Mean fluorescence of HEsEpiC cells stained with Prestoblue dye is shown for untreated controls, GO, and GO/COF composite at seeding densities of G) 50K and H) 100K cells/well.

The cytotoxicity evaluation of the GO/COF composite demonstrated excellent biocompatibility with human esophageal epithelial cells (HEsEpiC), supporting its potential use in medical applications. Due to the inherently short lifespan of primary esophageal epithelial cells in culture, passaged HEsEpiCs were used to ensure sufficient viability and consistency throughout the biocompatibility assays. Throughout the 7‐day incubation period, epithelial cells adhered consistently to the coated slides, showing robust proliferation with no adverse effects observed. Cell viability remained consistently high, exceeding 93.5% under all tested conditions, independent of initial seeding densities (50,000 or 100,000 cells/well). Importantly, no statistically significant differences (*p* > 0.05) in live cell percentages were found when comparing untreated controls, GO controls, and GO/COF composite samples across passages 4–6 (**Figure** **6**A–C). Interestingly, in Figure 6C, an increase in cell viability was observed in the GO/COF‐treated samples compared to controls. While the exact mechanism of this enhancement is unclear, the increased viability does not suggest cytotoxic effects. The live/dead staining assay revealed a negligible presence of dead cells, confirming that the GO/COF composite is non‐cytotoxic under the tested conditions (Figure 6D–F). These results were further corroborated by Prestoblue fluorescence assays (Figure 6G,H), which demonstrated comparable fluorescence intensity across all groups (*p* > 0.05). Notably, Prestoblue measurements were taken at multiple time points (2, 5, and 7 days), exceeding the anticipated monitoring timeline. The consistent absence of cytotoxicity over an extended period highlights the material's stability and suitability for longer‐term applications. These findings position the GO/COF composite as a promising material for use in biomedical devices, particularly in diagnosing GERD. Its ability to maintain high cell viability, even in prolonged exposure, indicates that it could be safely integrated into diagnostic platforms.

## 3 Conclusion

The combination of sulfonic acid‐functionalized COF with thermally reduced graphene oxide is proposed here to engineer a novel pH sensing material tailored for mucosa‐interfacing bioelectronics. Its integration onto soft, two‐ and three‐electrode platforms supports seamless adaptation to in vivo environments, meeting the demands of emerging flexible diagnostic technologies. The composite demonstrates high sensitivity in gastric acid simulants (≈43.5 mV pH^−1^, OCP), excellent acid stability, and high reproducibility—addressing critical challenges faced by existing electrochemical sensors in harsh sensing environments. The incorporation of ─SO_3_ groups within the COF nanochannels enabled a robust, reference‐free capacitive sensing mechanism confirmed by EIS, eliminating the need for traditional Ag/AgCl reference electrodes stabilized with Nafion, and thereby reducing cytotoxicity concerns. Furthermore, the material exhibited excellent biocompatibility with human esophageal epithelial cells and showed resistance to biofouling by mucin, bile salts, and digestive enzymes, reinforcing its suitability for prolonged *vivo* use. By overcoming the intrinsic conductivity limitations of COFs through thermally treated GO and simplifying sensor architecture without compromising performance, this study positions GO/sulfonic COF composites as a compelling alternative to conventional pH sensing materials. Their combined attributes of flexibility, biocompatibility, and scalable fabrication hold strong promise for real‐time gastrointestinal monitoring and broader applications in soft bioelectronics. Importantly, the GO/COF composite can be readily integrated into existing diagnostic platforms, such as ingestible capsules or implantable sensors. The sensor's electrochemical architecture is compatible with wireless readout mechanisms, including capacitive and impedance‐based approaches, which can be implemented via passive LC resonant circuits (e.g., NFC) or low‐power microelectronics. Furthermore, its low‐voltage operation can be supported by wireless power harvesting. Future efforts will focus on long‐term in vivo validation and integration into clinically relevant diagnostic platforms.

## 4 Experimental Section

### Materials

Silver/Silver Chloride (Ag/AgCl) paste (60:40 composition), Nafion 117 solution, graphene oxide aqueous solution (4 mg mL^−1^), octanoic acid, chloroform, N,N‐Dimethylformamide (DMF), p‐phenylenediamine (PDA), 2,5‐diaminobenzene‐1,4‐disulfonic acid (Pa‐SO_3_H), magnesium chloride (MgCl_2_), potassium chloride (KCl), calcium chloride (CaCl_2_), sodium chloride (NaCl), pepsin, bile salts, lipase and all other general solvents and reagents were obtained from Sigma Aldrich. SYLGARD 184 Polydimethylsiloxane (PDMS) was procured from Farnell. Gastric mucin was obtained from MedChemExpress. Silver screen‐printable ink (DM‐SIP‐2005) and carbon screen‐printable paste (DM‐CAP‐4311S) were sourced from Dycotec Materials. Ethanol was obtained from Fisher Scientific. Polyimide sheets (50 µm thickness) were purchased from Goodfellow Cambridge, and glass slides (75 mm × 50 mm) were from Clarity. 1,3,5‐Triformylphloroglucinol (TFP) was obtained from TCI Chemicals. 2,5‐Diaminobenzoic acid was sourced from Lab Pro Inc. Human Esophageal Epithelial Cells (HEsEpiC) (Ref: P60152), poly‐L‐lysine, and epithelial cell medium were purchased from Innoprot. Six‐well culture plates were obtained from Costar. The LIVE/DEAD Viability/Cytotoxicity Kit (L3224), PrestoBlue and PrestoBlue HS Cell Viability Reagents (A13262) were purchased from ThermoFisher Scientific. Phosphate‐buffered saline was sourced from Gibco (ThermoFisher 18912‐014). All chemicals were used as received without further purification.

### Flexible, 3‐Electrode Sensor Fabrication

The three‐electrode design was screen‐printed onto a polyimide sheet, with each ink layer dried and cured per manufacturer's guidelines. First, DM‐SIP‐2005 formed the silver conductive tracks, followed by DM‐CAP‐4311S for the working and counter electrodes. The sensor was then transferred to a direct‐write printer with a fixed syringe and an XYZ Aerotech Nanopositioner (ANT130L and ANT130XY) to print the PDMS dielectric encapsulation layer, chosen for its flexibility and biocompatibility (Figure S1, Supporting Information). A heated stage enabled in situ curing of the thermally cured PDMS.^[^ ^45^, ^46^ ^]^

### Synthesis of GO/COF Composite

Briefly, 10 mg of GO from aqueous solution was freeze‐dried for 2 days to remove the solvent and stored in glass vials. 10 mL of DMF was added to the glass vial and bath sonicated in an ice bath for 2 h to ensure uniform dispersion. 10 mg of TFP was then added to the glass vial and bath sonicated for 10 min. A glass slide was put on top of a hot plate heated to 80°C followed by drop casting the mixture solution on the glass. 10 mL of octanoic acid was poured into a separate beaker and 10 mg of the second linker (i.e., Pa‐SO_3_H, 2,5‐diaminobenzoic acid, or PDA) was added to the octanoic acid, followed by bath sonication for 30 min. The glass slide with GO and linker was then added (as shown in Figure S1, Supporting Information) to allow for solid‐vapor interfacial vaporization as described by our previous work.^[^ ^34^ ^]^ The beaker was wrapped with aluminum foil to prevent the escape of the vapor, followed by placing in an oven at 150°C for 12 h. Subsequently, the as‐formed GO/COF film was washed intensely with chloroform to remove impurities and excess reactants and peeled from the glass. The film was then put into a glass vial containing 10 mL of DMF and bath sonicated for 2 h for liquid exfoliation. This step crucially allowed for thinner layers of GO/COF nanosheets to ensure greater availability of active sites. The solution was then centrifuged and washed with chloroform 3 times. The chloroform solution was sonicated to uniformly disperse the GO/COF in the solution and prepare for COF activation.^[^ ^47^ ^]^ The solution was then drop cast onto the working electrode area of the sensor, followed by placing the sensor in a vacuum oven for 12 h at 80°C to remove the remaining solvent to complete COF activation. Unless otherwise specified, all GO/COF composites were synthesised using a mass ratio of 1:1 for TFP to GO.

### Material Characterization

To characterize the formation of COF, Fourier‐Transform Infrared spectroscopy (FTIR) was utilized using a Spectrum Two, Perkin Elmer spectrophotometer in the region of 400 to 4000 cm^−1^. The samples were peeled from the glass and placed onto the laser to minimize interference during the spectroscopy. Analysis of peaks was performed using MATLAB 2023b. The surface morphologies were imaged using scanning electron microscopy (SEM) (ZEISS GeminiSEM 360). All COFs were activated prior to imaging and were applied onto a metal stub with double‐sided adhesive carbon tape. A thin gold film was sputter coated for improved electron microscopy quality. Energy dispersive X‐ray spectroscopy (EDS) was also performed to produce elemental composition maps. The GO/COFs were imaged in three different configurations, 1) following peeling from the glass to obtain thin films, 2) following liquid exfoliation, solvent transfer and COF activation stages detailed in Figure S2 (Supporting Information), and 3) following immersion in a protein‐rich environment for 24 h. X‐ray diffraction (XRD) measurements were performed on Malvern Panalytical Aeris X‐ray diffractometer. Atomic force microscopy (AFM) was also performed using Bruker MultiMode 8 with ScanAsyst probes on the dispersed sample. Surface roughness (Ra) values were extracted from the AFM height images using the NanoScope Analysis software to quantitatively compare the surface morphology of GO and GO/COF samples. An in‐house goniometer setup was used for performing wettability and contact angle measurements. The setup consists of an adjustable stage, laboratory stand, syringe pump (World Precision Instruments, Aladdin single‐syringe infusion pump), light source (Thorlabs, OSL2) and a magnification lens (Thorlabs, MVL7000) fitted to a camera. Videos were recorded to measure the advancing angles, and analysis was performed using ImageJ software^27^. To assess the mechanical durability of the flexible sensor, cyclic compression‐induced bending tests were performed using an Instron universal testing machine (model 5969). The sensor (15 mm in length, 0.06 mm in thickness measured by digital vernier caliper) was mounted in a double‐clamped configuration, and subjected to axial displacement‐controlled compression at a rate of 25 mm min^−1^. A total compressive displacement of 0.5 mm was applied in each cycle, inducing a bending curvature corresponding to a maximum surface strain of ≈0.3%. The test was repeated for 1000 cycles at room temperature under ambient conditions, and the force–displacement response was recorded continuously. CV measurements were conducted before and after the bending test to monitor changes in redox behavior and electrode integrity.

### Cytotoxicity Assessment

HEsEpiC cells were used to assess cytotoxicity with GO/COF composites compared against pristine GO as a control due to well documented low cytotoxicity and good biocompatibility in literature. All experiments were performed between passages 2–5. Cytotoxicity was assessed using a live/dead viability assay following a 7‐day incubation period. The assay utilized fluorescent dyes, calcein‐AM and ethidium homodimer‐1, to stain live and dead cells, respectively. The staining solution was prepared by adding 5 µL of calcein‐AM (Component A) and 20 µL of ethidium homodimer‐1 (Component B) to 10 mL of PBS, ensuring thorough mixing for homogeneity. This procedure was conducted using the L3224 Live/Dead Viability/Cytotoxicity Kit, strictly adhering to the manufacturer's instructions. The prepared dye solution was applied to the cell culture wells and incubated for 30 min at room temperature (20–25°C). Fluorescence microscopy (Axio Observer Zeiss microscope) was employed to visualize and quantify the cells, with excitation/emission wavelengths of 494/517 nm for calcein‐AM (green fluorescence for live cells) and 528/617 nm for ethidium homodimer‐1 (red fluorescence for dead cells). An automated image analysis program was developed for accelerating the quantification of cells (Figures S11 and S12, Supporting Information).

Cell metabolic activity was assessed using PrestoBlue and PrestoBlue HS Cell Viability Reagents. High fluorescence intensity indicated metabolically active living cells, while low fluorescence intensity corresponded to dead or metabolically inactive cells. PrestoBlue was diluted 1:10 in culture medium, and 400 µL of the solution was added to each well of the cell culture plate. The plate was then incubated for 30 min at room temperature (20–25°C). Technical triplicates were prepared, with 100 µL from each well transferred to a black‐walled plate for measurement. Fluorescence intensity was recorded using a plate reader (Tecan Infinite 200 Pro) at excitation and emission wavelengths of 560 and 590 nm, respectively. Background fluorescence was determined using PrestoBlue without cells and subtracted from the experimental readings to calculate net fluorescence. Further details on cell culturing, and control samples preparation procedure details are in the Supporting Information.

### Statistical Analysis

All data are expressed as the mean ± standard deviation from three independent experiments/samples. Pairwise comparisons between groups were conducted using independent *t*‐tests, and a p‐value of less than 0.05 was considered statistically significant.

## Conflict of Interest

The authors declare no conflict of interest.

## Supporting information



Supporting Information

## Data Availability

The data that support the findings of this study are available from the corresponding author upon reasonable request.
