# Supplementary material for: Robust pH Sensing Using a Graphene Oxide and Covalent Organic Frameworks Composite for Gastro‐esophageal Reflux Disease Diagnosis
Source: Adv Healthc Mater. 2025 Aug 5;14(32):e02106. doi: 10.1002/adhm.202502106 (PMC12716212; doi:10.1002/adhm.202502106)
Supplement: Supplementary file 1 — Supporting Information [file ADHM-14-0-s001.docx]

Robust pH sensing using a Graphene Oxide and Covalent Organic Frameworks Composite for Gastro-esophageal Reflux Disease Diagnosis

Ahmed H. Salem^1,2,3^, ^‡^ Jianhui Zhang^1,2^, ^‡^ Ashley Lam^4^, Haowei Wang^5^, Umber Cheema^4^, Laurence B. Lovat^2^, Manish K. Tiwari^1,2,6 *^

^1^Nanoengineered Systems Laboratory, UCL Mechanical Engineering, University College London, London, WC1E 7JE, UK.

^2^UCL Hawkes Institute, University College London, London, W1W 7TS, UK.

^3^UCL Medical Physics and Biomedical Engineering, University College London, London, WC1E 7JE, UK.

^4^UCL Centre for 3D Models of Health and Disease, UCL Division of Surgery and Interventional Science, Faculty of Medical Sciences, Charles Bell House, University College London, London W1W 7TY, UK.

^5^Centre for Precision Healthcare, UCL Division of Medicine, University College London, London, WC1E 6JF, UK

^6^Manufacturing Futures Laboratory, University College London, London, E20 2AE, UK

‡A.H.S. and J.Z. contributed equally.

**Corresponding author email:** m.tiwari@ucl.ac.uk

Supporting Information

# Cytotoxicity Testing Methods

Control Samples and Preparation

GO was selected as the control material due to its extensively documented cytotoxicity and biocompatibility. Glass squares (1 cm × 1 cm) were cleaned using acetone, isopropyl alcohol, and deionized water (all procured from Sigma Aldrich). Oxygen plasma treatment (Diener Femto device) was performed at 0.1 mBar and 30 W for 4 minutes to further purify the surfaces. Ethanol rinsing followed to minimize microbial contamination and static charge from the plasma treatment.

For coating, solutions of GO and the GO/COF composite were prepared at equal concentrations (0.1 mg/mL), dispersed in water and chloroform, respectively. Each glass slide was coated by drop-casting 40 µL of the respective solution, followed by air-drying. The GO/COF-coated slides were further treated in a vacuum oven at 80 °C for 12 hours to remove residual chloroform from nanopores and activate the COF.

Cell Culture, Seeding and Passage

All experiments were performed between passages 2-5 on HEsEpiC cells. Cytotoxicity tests were conducted using HEsEpiC cells at passages 2-5 to ensure both biological relevance and experimental reproducibility. Early-passage cells retain key epithelial characteristics representative of native esophageal tissue, while avoiding the variability and limited lifespan of primary cultures. This range also ensures stable growth and viability over the assay duration, which is critical for reliable toxicity assessment. Pre-coating of six-well cell culture plates was performed using poly-L-lysine (2 μg/cm²) to enhance HEsEpiC cell attachment. The plates were incubated overnight at room temperature in the poly-L-lysine solution, after which the coating solution was removed, and the plasticware was washed three times with sterile water. The coated plasticware was then sealed with Parafilm and stored at 4 °C for up to two weeks prior to cell seeding.

Glass samples were prepared using the same poly-L-lysine coating procedure before cell seeding. HEsEpiC cells were then seeded at densities of 50,000 or 100,000 cells per well (6-well plate) in epithelial cell medium for seven days. Half-volume medium changes were performed every two to three days by replacing half of the medium in each well.

For routine maintenance, HEsEpiC cells underwent half-volume medium changes every two days and were passaged at a 1:5 ratio every five days. During passaging, the cells were detached using a solution prepared by diluting TrypLE Express Enzyme 1:1 with PBS. The cells were incubated in this detachment solution at 37 °C for three to five minutes, after which the detached cells were collected. The TrypLE solution was neutralized by adding twice the volume of cell culture medium, and the cell pellet was recovered by centrifugation. All cultures were maintained in a humidified incubator at 37 °C with 5% CO_2_.

Image Analysis and Quantification

Cell viability was calculated as the percentage of live cells (calcein-AM positive) relative to the total cell population. An automated image analysis protocol was developed using MATLAB-2023a® for high-throughput quantification (Figure S11 and S12). Cellular images were converted into binary maps using thresholding to distinguish cells from the background. A seeded watershed-based algorithm was applied to identify cell boundaries, with over-segmentation corrected using the MATLAB Image Processing Toolbox. The protocol, validated against manual counting, achieved >99% correlation, ensuring reliable and reproducible results. Based off ^[1]^.

Supporting Figures


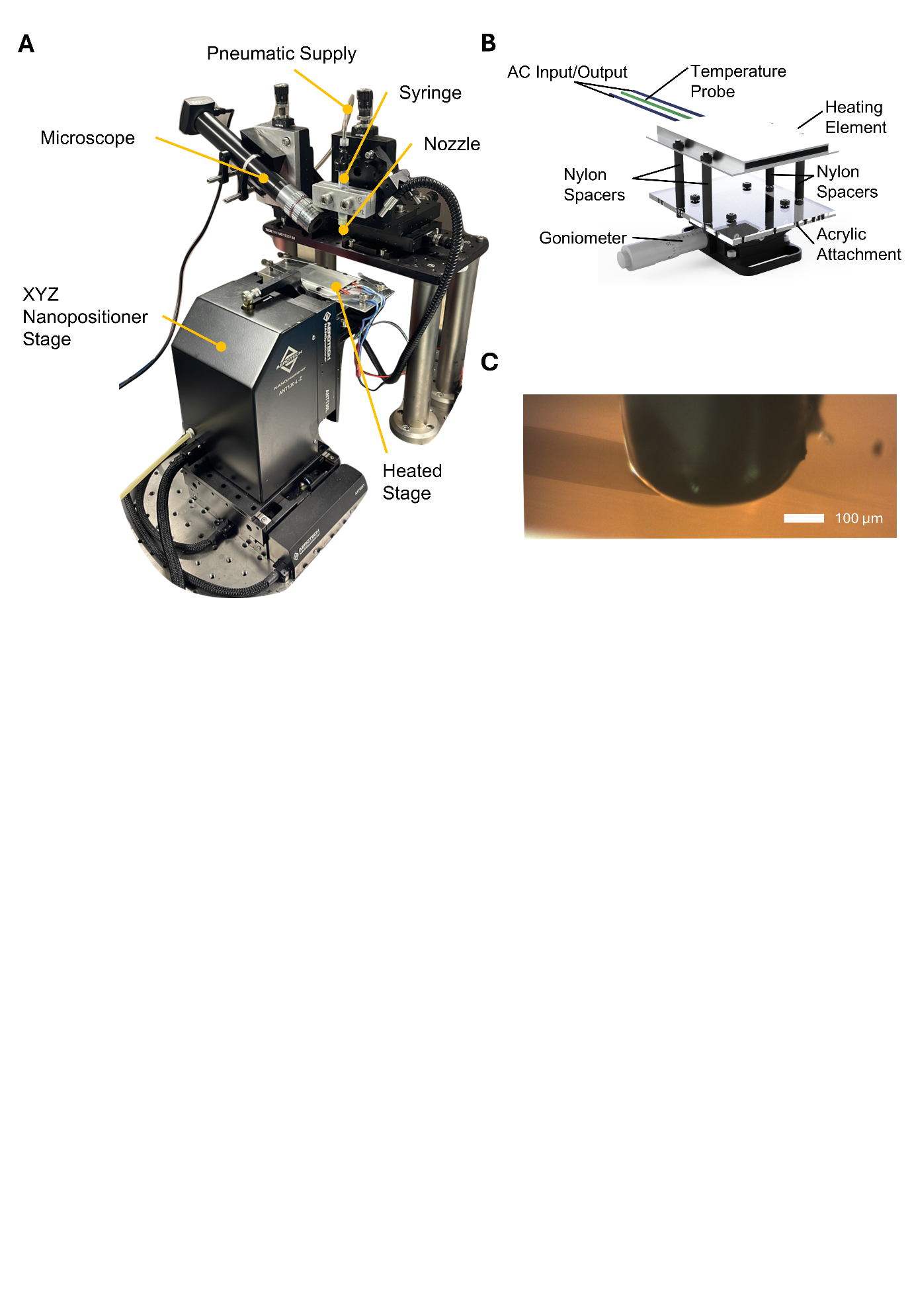


**Figure S1.** (A) Diagram of direct-write printing setup used for sensor fabrication (B) Heated stage setup schematic used to maintain nozzle-substrate distance and in situ curing of PDMS (C) Microscope image of PDMS printing for biocompatible encapsulation.


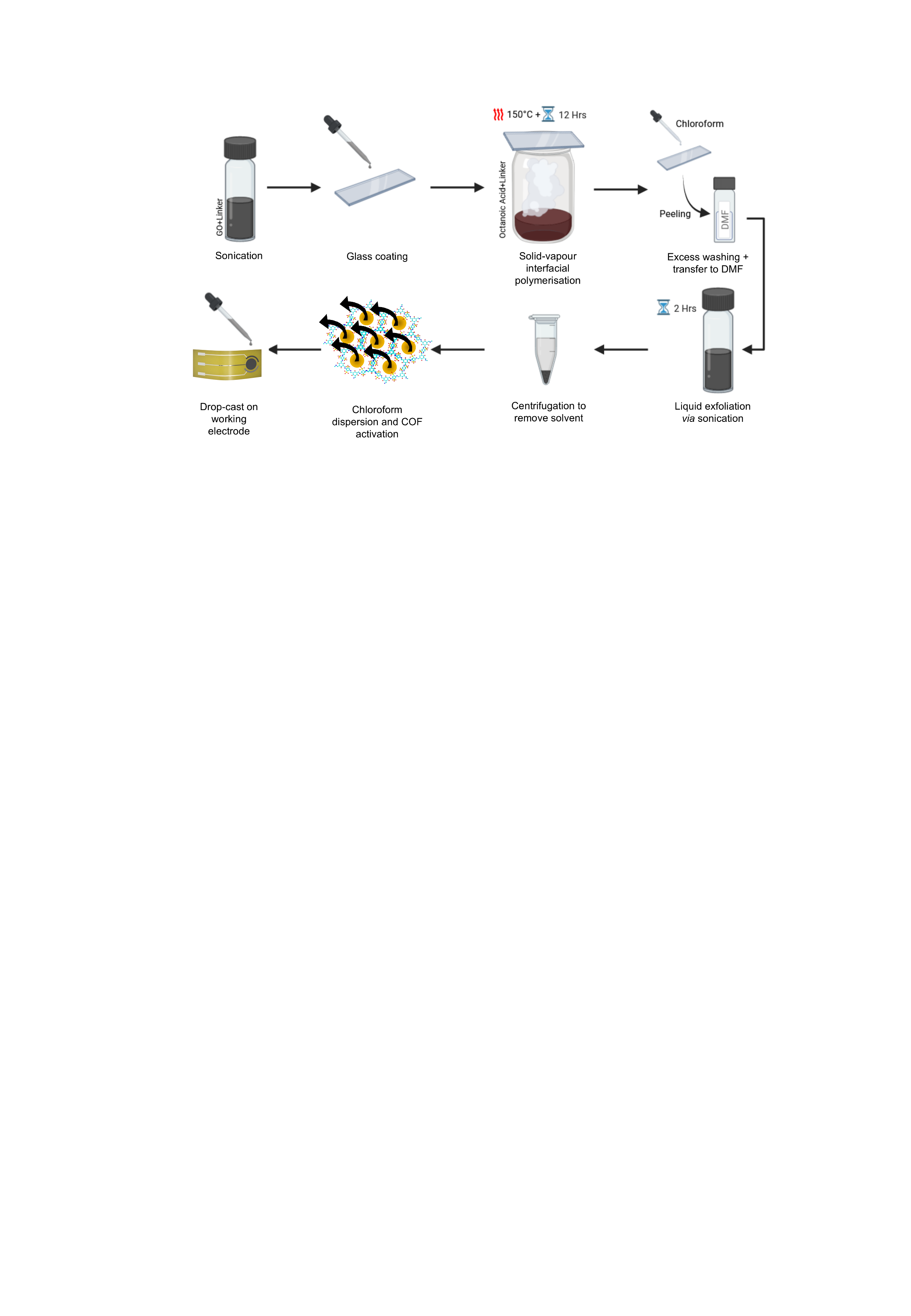


**Figure S2.** Schematic of method used for synthesizing different types of COFs.


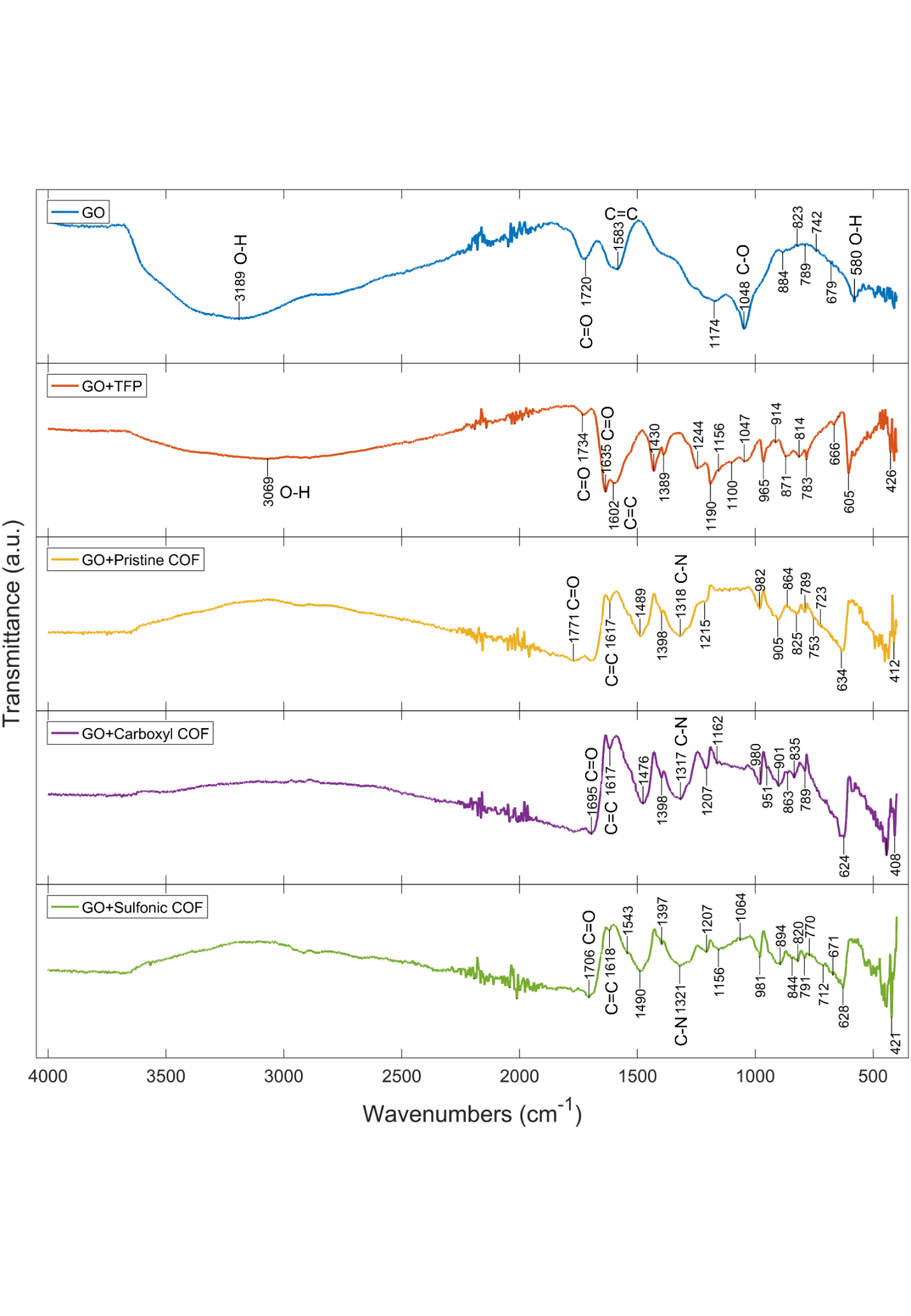


**Figure S3.** FTIR spectra of graphene oxide (GO), the GO-TFP reaction intermediate, and GO/COF composites with different surface functionalities: pristine, carboxylated, and sulfonated. Peaks marked according to ^[2–5]^.


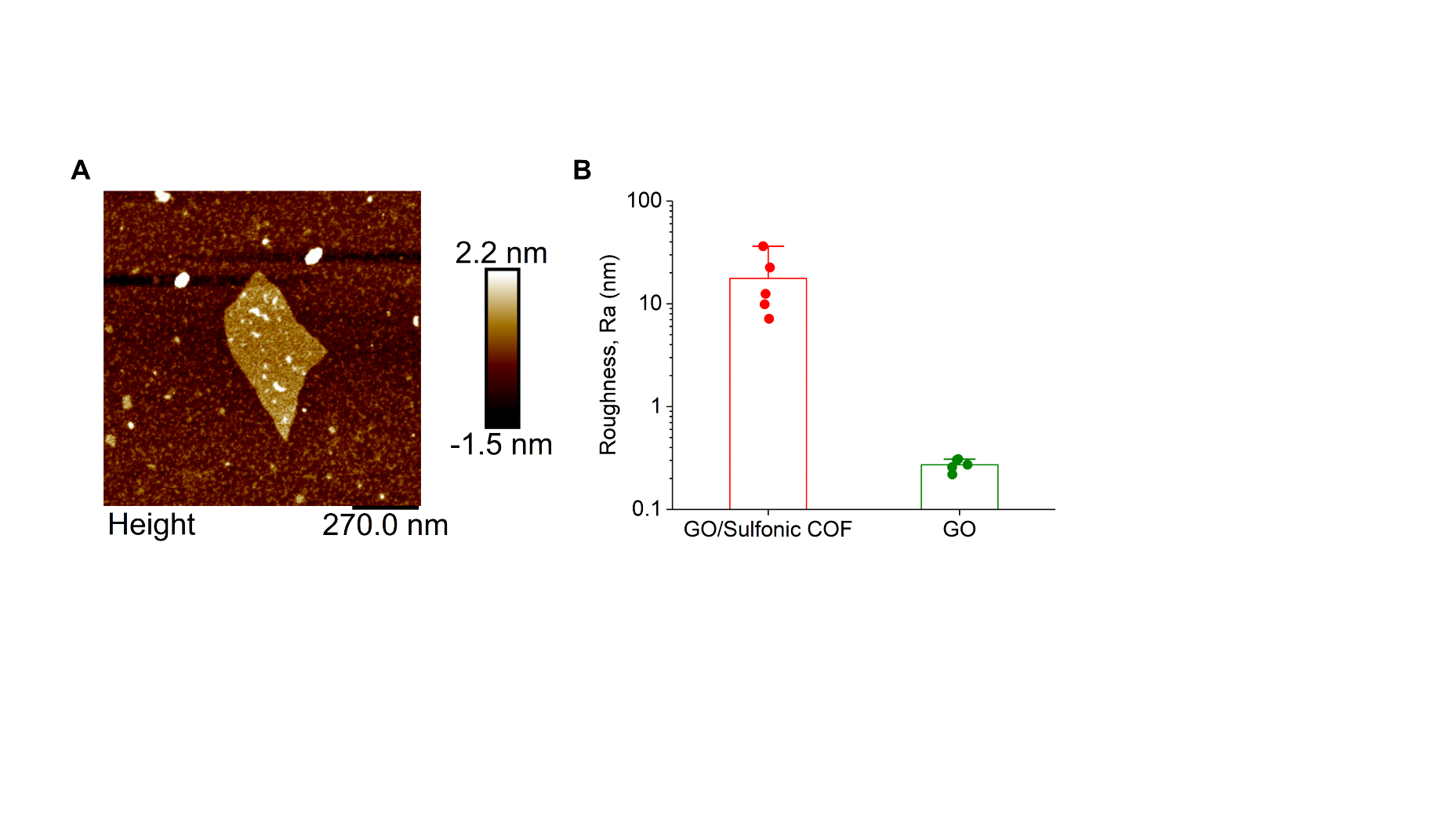
**Figure S4.** (A) AFM images on single GO sheet and (B) Surface roughness of GO/COF and GO, quantified through AFM scanning.


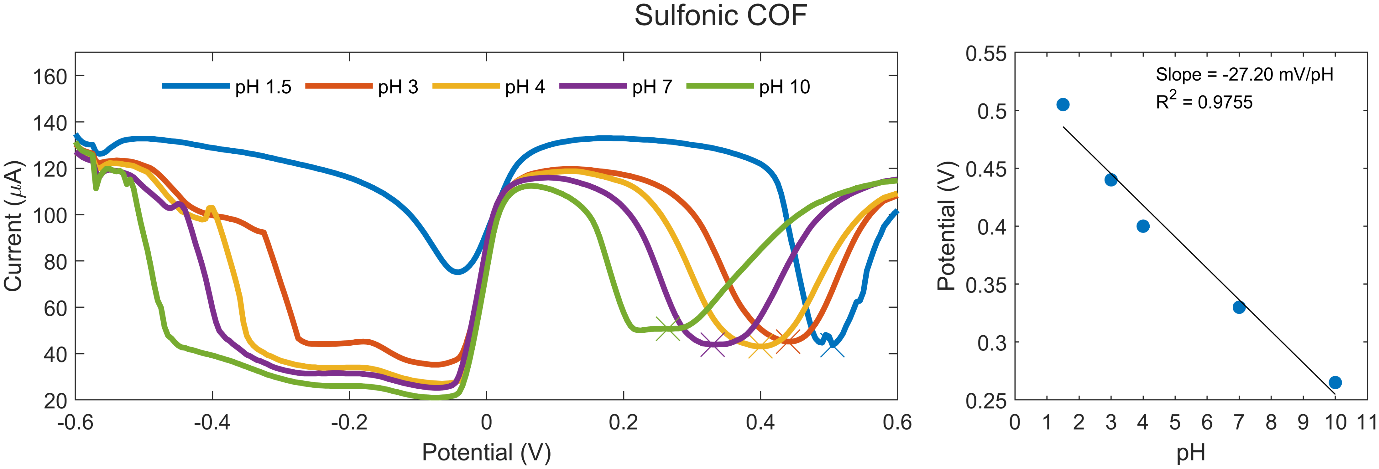


**Figure S5.** Differential Pulse Voltammetry (DPV) plot and corresponding calibration curve against pH revealing high linearity and sensitivity.


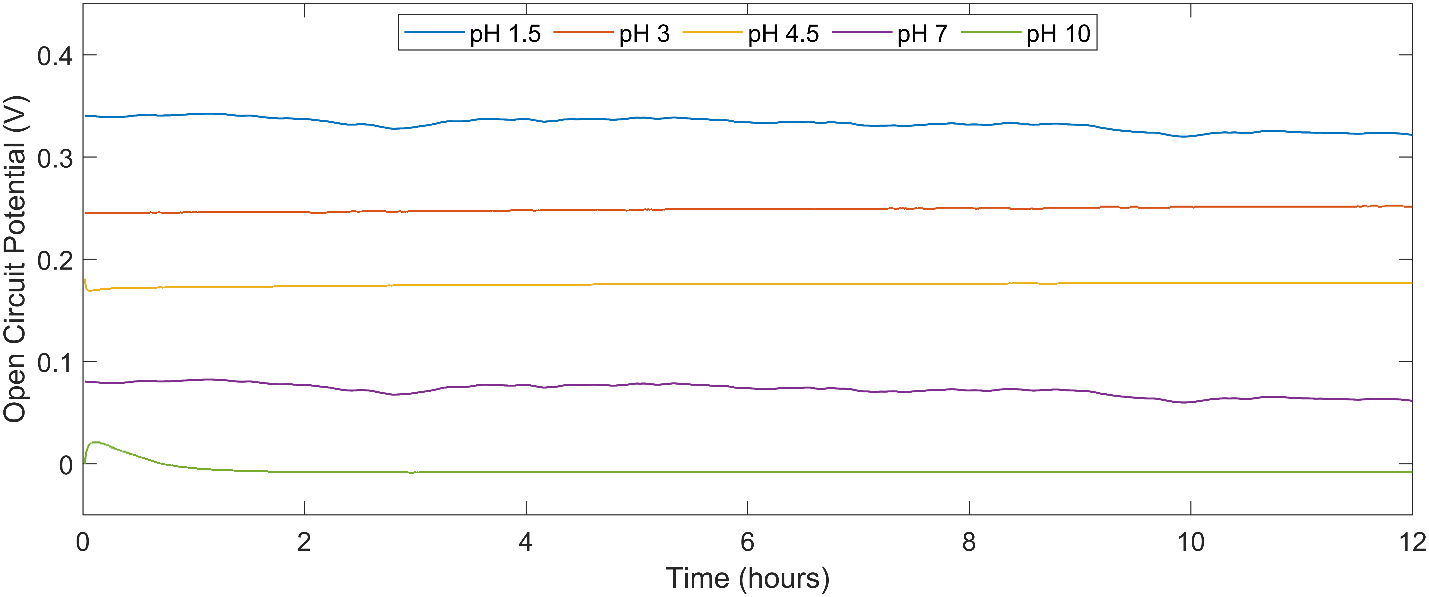


**Figure S6.** Open-circuit potential (OCP) stability of the GO/COF sensor over 12 hours at various pH conditions (pH 1.5, 3, 4.5, 7, and 10)


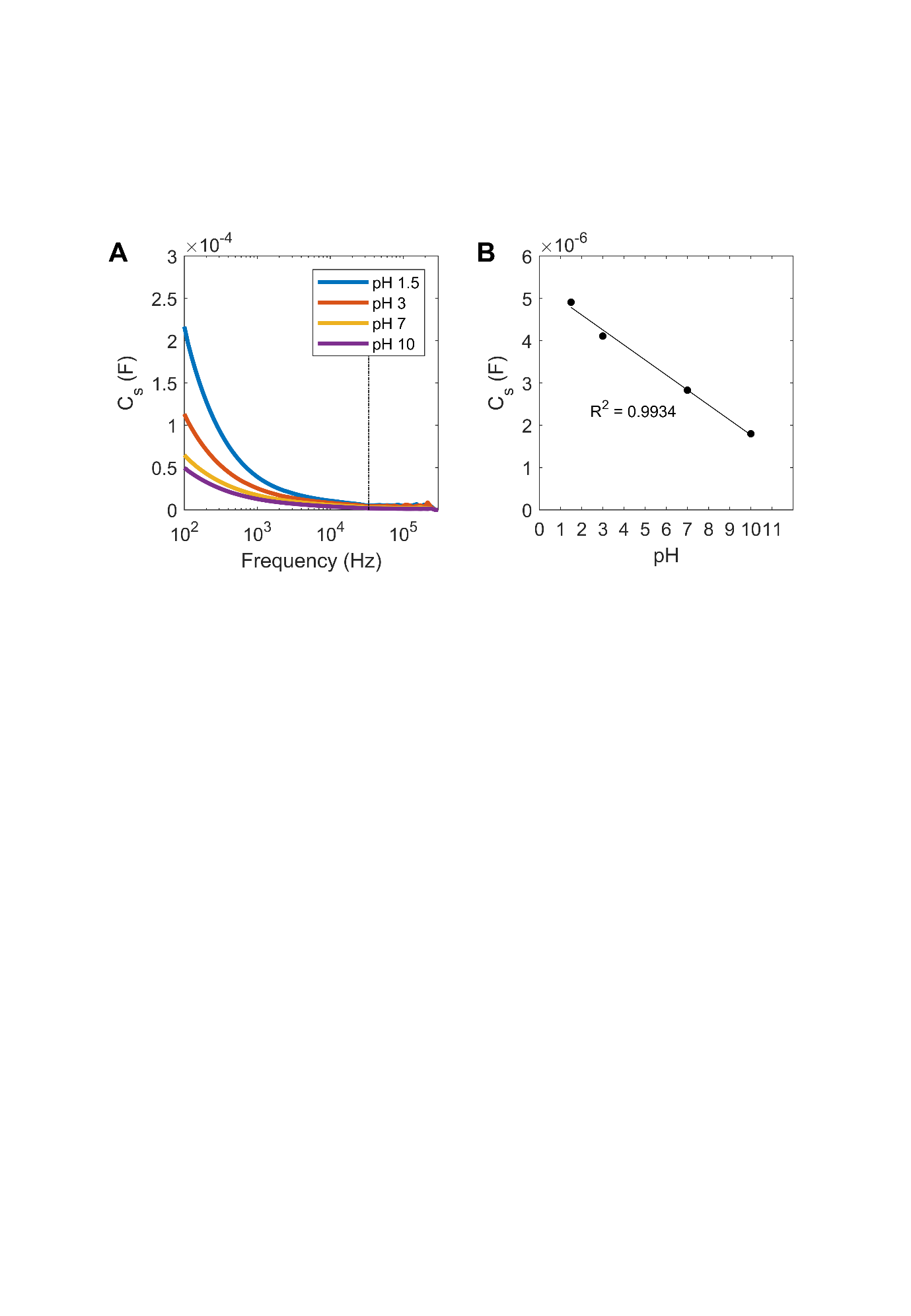


**Figure S7****.** Capacitance response of the Sulfonic COF and GO composite in pH solutions, demonstrating that protons are the primary factor influencing the electrical double layer capacitance. (B) Calibration curve of capacitance to pH at indicated dashed line shown in (A).


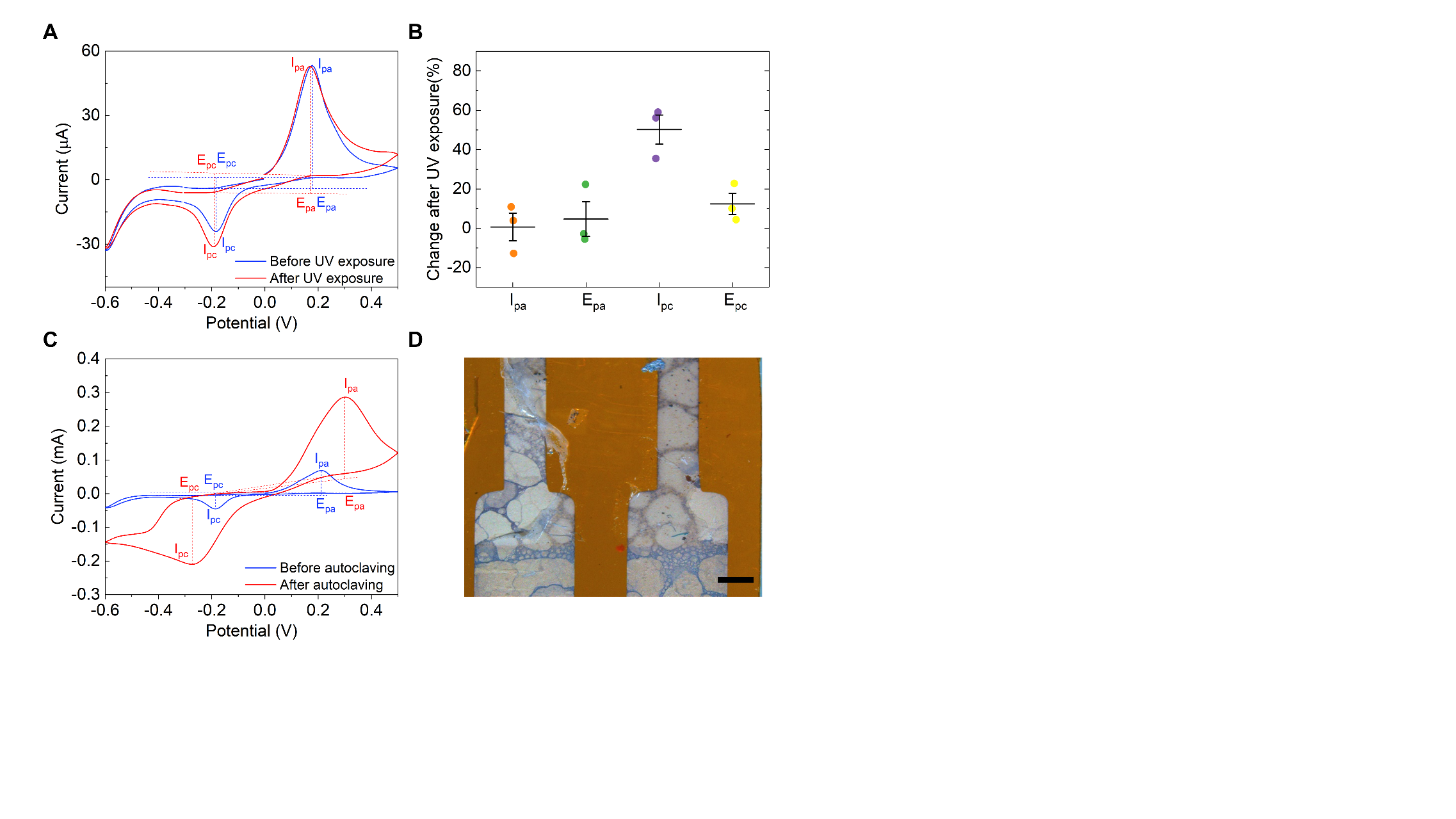


**Figure S8.** Evaluation of sensor stability after clinical sterilisation procedures. (A) CV curves of the sensor before and after UV sterilisation, showing minimal change in electrochemical behaviour. (B) Corresponding peak currents and potentials, confirming stable performance post-UV exposure. (C) CV curves before and after autoclaving, indicating a significant increase in current response. (D) Optical image of the sensor after autoclaving, showing partial delamination of the PDMS encapsulation layer and subsequent crack formation. Scale bar: 500 µm.


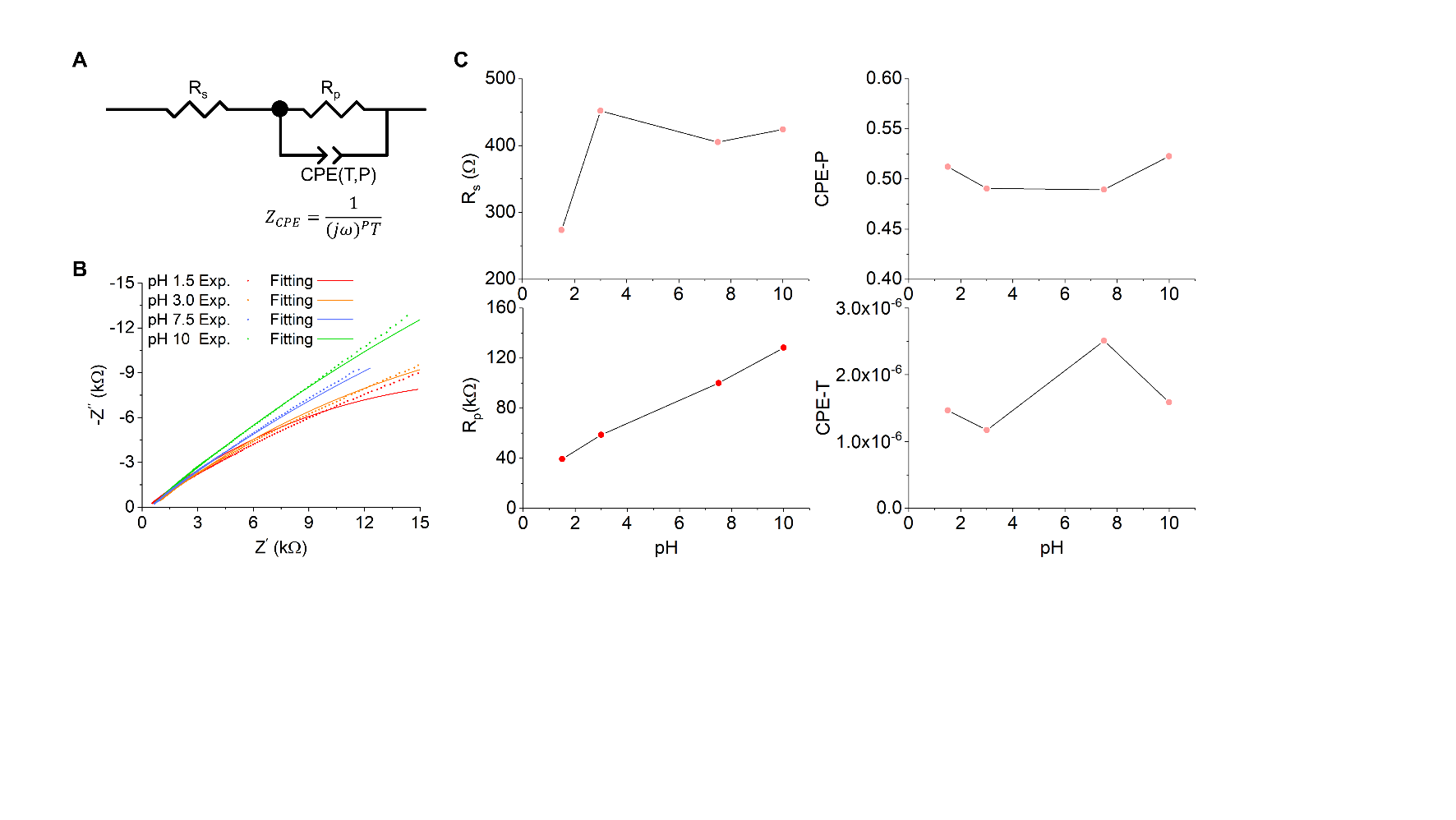


**Figure S9.** Electrochemical impedance spectroscopy (EIS) analysis of the sensor in solutions of varying pH, and fitted parameters based on the equivalent circuit model. (A) Equivalent circuit used to fit the EIS data, consisting of a solution resistance (R_s_), a polarization resistance (R_p_), and a constant phase element (CPE) to account for non-ideal capacitive behavior. In the CPE, T is a pseudo-capacitance factor and P is an empirical exponent. (B) Nyquist plots showing the measured real (Z') and imaginary (Z'') components of impedance (data points), overlaid with fitted curves based on the equivalent circuit model. (C) Extracted circuit parameters (R_s_, R_p_, CPE-T and CPE-P) as a function of pH, indicating the sensor’s pH-dependent electrochemical behavior.


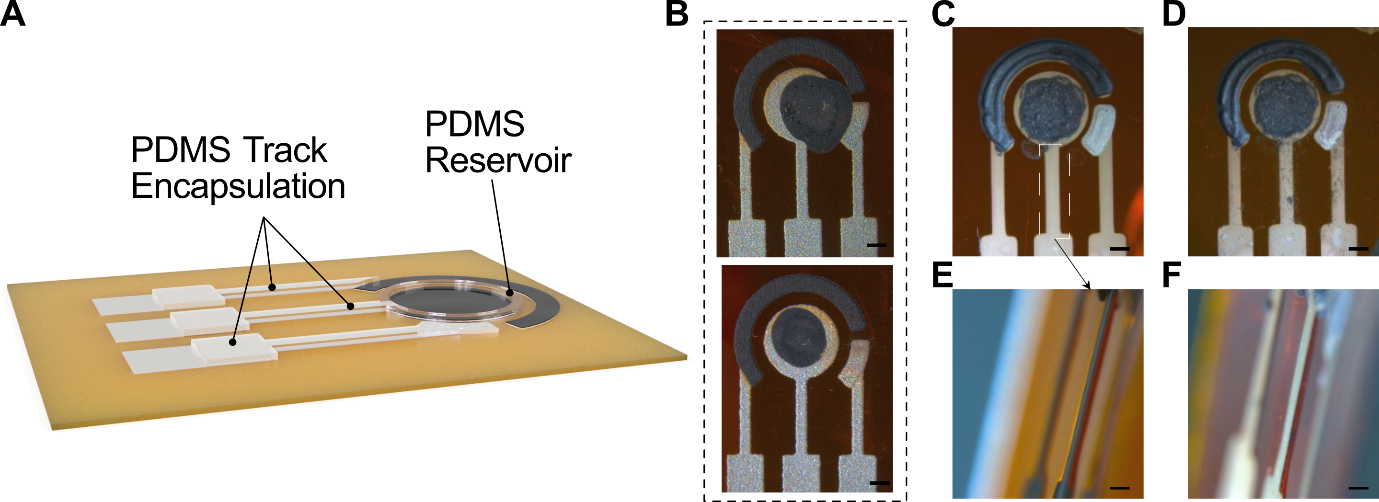


**Figure S10.** PDMS encapsulation preserves structural integrity and electrode performance under mechanical stress. (A) Schematic of the PDMS encapsulation. (B) Incomplete WE coverage caused by reservoir removal, shown via optical images. (C, D) Top-view optical images of the sensor’s three electrodes with PDMS reservoir before (C) and after (D) 1000 bending cycles, showing no visible damage to the working, reference, or counter electrodes. (E, F) Side-view images of the PDMS-encapsulated silver interconnects before (E) and after (F) bending, with no signs of delamination or cracking. Scale bars: 0.6 mm.


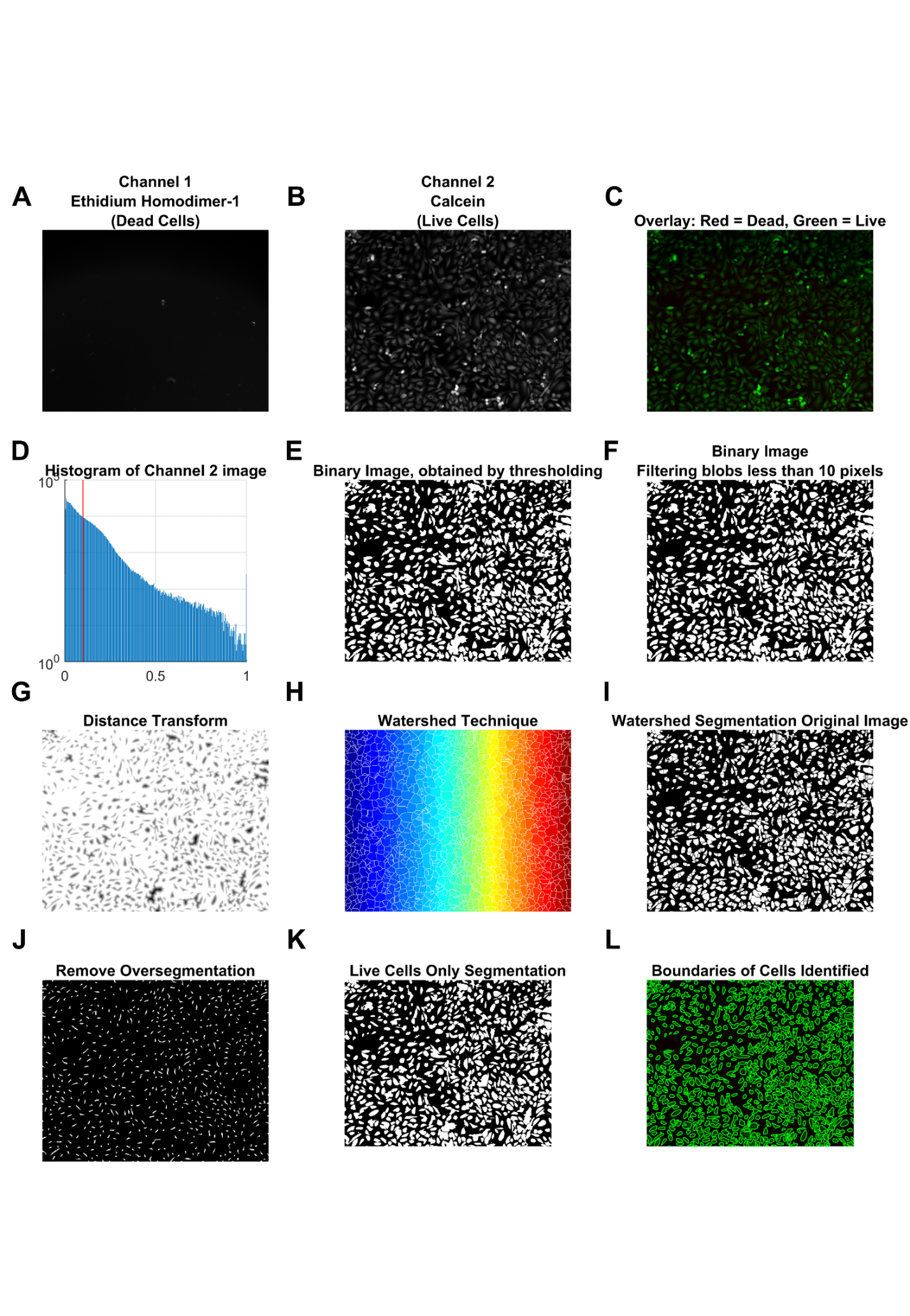


**Figure S11. (**A) Channel 1 of the microscope file showing the Dead Cells (B) Channel 2 showing the live cells in grayscale image (C) Overlay of the channels showing their original colors. (D) Histogram of Channel 2 image used for thresholding the Binary image shown in (E). (F) Applying filters to remove noisy data less than 10 pixels in size. (G) Applying a distance transform performed by MATLAB image processing toolkit. (H) Applying the watershed technique to identify the boundaries of the cells. (I) Using the identified boundaries to segment the original image. (J) Removing oversegmentation by identifying minimum segmented distance (K) Image following full segmentation (L) Highlighted boundaries of live cells identified by function (shown in green).


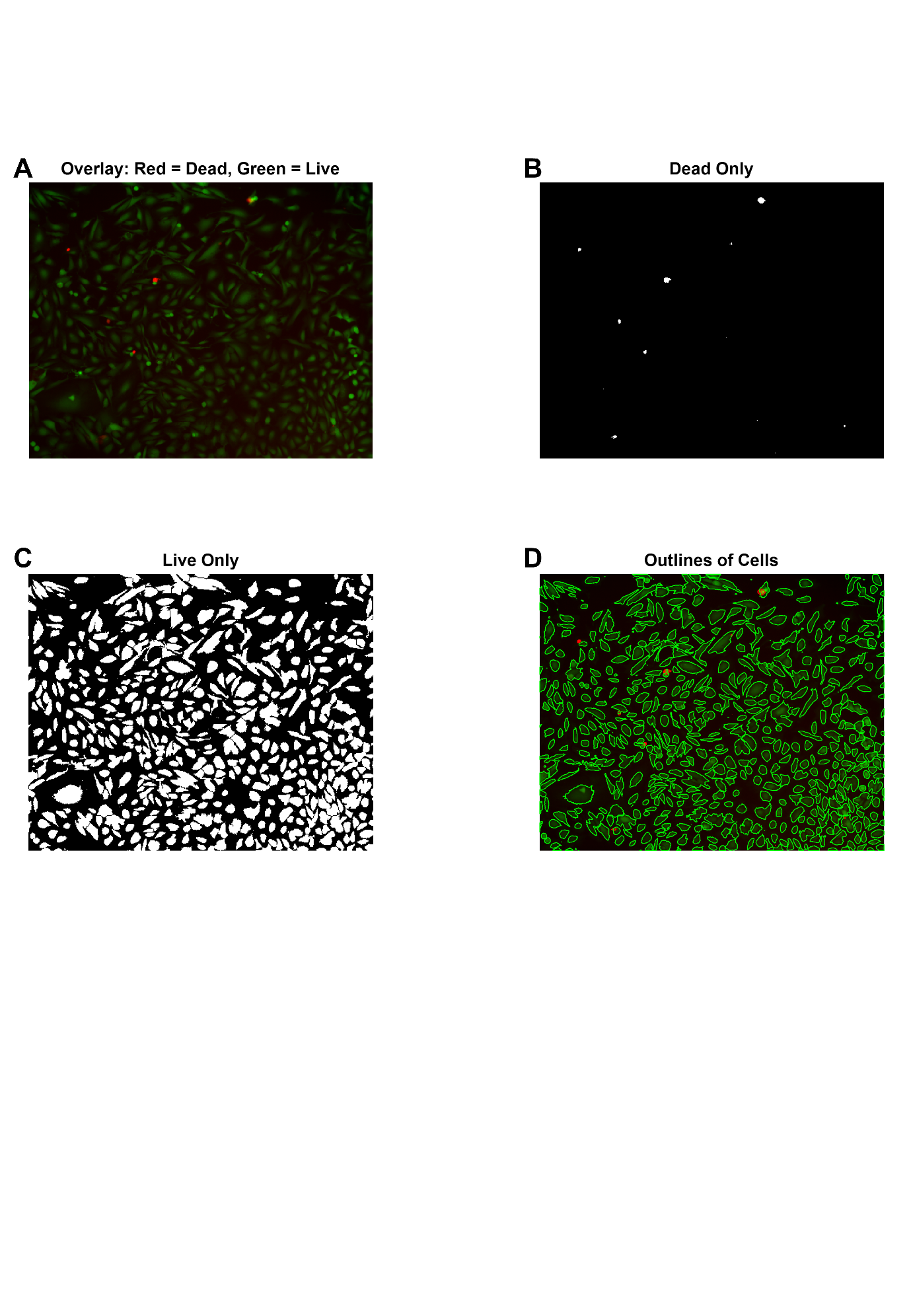


**Figure S12.** Representative example of post-processed imaging to differentiate live and dead cells. (A) Original image with overlaid colors for context. (B) Channel 1: Processed image highlighting dead cells. (C) Channel 2: Processed image highlighting live cells. (D) Segmented boundaries of live cells (green) and dead cells (red) for enhanced visualization.

# References

[1] Eddins, S. Marker-Controlled Watershed Segmentation, 2013. https://uk.mathworks.com/help/images/marker-controlled-watershed-segmentation.html.

[2] Peng, Y., Xu, G., Hu, Z., et al., **2016**, *ACS Applied Materials & Interfaces*, *8*, 18505, 10.1021/acsami.6b06189.

[3] Kandambeth, S., Mallick, A., Lukose, B., Mane, M. V., Heine, T., Banerjee, R., **2012**, *Journal of the American Chemical Society*, *134*, 19524, 10.1021/ja308278w.

[4] Singh, V., Zhang, J., Chen, J., Salzmann, C. G., Tiwari, M. K., **2023**, *Advanced Materials*, *35*, 2302466, 10.1002/adma.202302466.

[5] InstaNANO. FTIR Functional Group Database Table with Search. https://instanano.com/all/characterization/ftir/ftir-functional-group-search/ (accessed 2025-04-02).
